# Structural Insights into the FtsQ/FtsB/FtsL Complex, a Key Component of the Divisome

**DOI:** 10.1038/s41598-018-36001-2

**Published:** 2018-12-24

**Authors:** Yuri Choi, Jinwoo Kim, Hye-Jin Yoon, Kyeong Sik Jin, Sangryeol Ryu, Hyung Ho Lee

**Affiliations:** 1 Department of Chemistry, College of Natural Sciences, Seoul National University, Seoul, 08826 Korea; 2 Department of Food and Animal Biotechnology, and Research Institute for Agriculture and Life Sciences, Seoul National University, Seoul, 08826 Korea; 3 Department of Agricultural Biotechnology, Seoul National University, Seoul, 08826 Korea; 4 Pohang Accelerator Laboratory, Pohang University of Science and Technology, 80 Jigokro-127-beongil, Nam-Gu, Pohang, Kyungbuk 37673 Korea; 5 Center for Food and Bioconvergence, Seoul National University, Seoul, 08826 Korea

## Abstract

Bacterial cell division is a fundamental process that results in the physical separation of a mother cell into two daughter cells and involves a set of proteins known as the divisome. Among them, the FtsQ/FtsB/FtsL complex was known as a scaffold protein complex, but its overall structure and exact function is not precisely known. In this study, we have determined the crystal structure of the periplasmic domain of FtsQ in complex with the C-terminal fragment of FtsB, and showed that the C-terminal region of FtsB is a key binding region of FtsQ via mutational analysis *in vitro* and *in vivo*. We also obtained the solution structure of the periplasmic FtsQ/FtsB/FtsL complex by small angle X-ray scattering (SAXS), which reveals its structural organization. Interestingly, the SAXS and analytical gel filtration data showed that the FtsQ/FtsB/FtsL complex forms a 2:2:2 heterohexameric assembly in solution with the “Y” shape. Based on the model, the N-terminal directions of FtsQ and the FtsB/FtsL complex should be opposite, suggesting that the Y-shaped FtsQ/FtsB/FtsL complex might fit well into the curved membrane for membrane anchoring.

## Introduction

Bacterial cell division is a fundamental process that results in the physical separation of a mother cell into two daughter cells and involves a set of proteins known as the divisome. The divisome of Gram-negative bacteria consists of more than 30 proteins localized to the mid-cell, which include various proteins (FtsA, FtsB, FtsI, FtsK, FtsL, FtsN, FtsQ, FtsW, FtsZ, and ZipA), and is responsible for complex and coordinated remodeling of the three-layered cell envelope, where membrane fusion will be induced^1,2^. Recent studies purified a 1-MDa protein complex of the divisome containing critical proteins, suggesting that the divisome is assembled in a highly coordinated fashion^3^ (Supplementary Fig. S1A). Some divisome proteins are essential for bacterial viability; thus, exploiting these proteins would be useful in discovering antibiotics that curb bacterial growth^4^.

Assembly and disassembly of the divisome occurs for each round of cell division in a predominantly hierarchical order. Assembly of the divisome is a two-step process; first, an organized polymer known as the FtsZ ring (Z-ring), which provides constrictive force, is anchored to the inner surface of the cytoplasmic membrane by early components including FtsA and ZipA^4,5^. Next, late proteins including FtsK, the FtsQBL complex (FtsQ, FtsB, and FtsL), FtsW, FtsI, and FtsN are recruited^6,7^. FtsK, a DNA translocase, unlinks chromosome dimers^8^, whereas FtsW is involved in septal peptidoglycan synthesis, with a proposed role of a peptidoglycan glycosyltransferase by binding to penicillin-binding protein such as FtsI^9–12^. FtsN is responsible for the recruitment of two septal components (the murein hydrolase AmiC and the Tol-Pal complex) and also induces a conformational switch in FtsA and the FtsQBL complex, so that septal peptidoglycan synthesis and membrane invagination can be de-repressed^13–15^ (Supplementary Fig. S1B).

Early and late components are linked by the FtsQBL complex, which is well-conserved among bacteria^16^. In expanding the molecular understanding of the FtsQBL complex, its structural organization has been explored and several intensive biochemical studies have been performed by several research groups. The *E. coli* FtsQBL complex was isolated by co-immunoprecipitation^17^ and the physical interactions between its components were detected by bacterial two-hybrid analysis^18^. Crystal structure of the periplasmic domains of *E. coli* and *Yersinia enterecolitica* FtsQ was solved, revealing partial structural information about the FtsQBL complex^19^. Interestingly, the periplasmic subcomplex of FtsQBL without the N-terminal membrane regions of FtsQ, FtsB, and FtsL was reconstituted *in vitro* by replacing the membrane parts with oppositely charged soluble coiled coils, building a structural model of a 1:1:1 FtsQBL complex in solution^20–22^. A mass spectrometric analysis by photo cross-linking indicated that the C-terminal regions of FtsQ and FtsB are crucial interaction hotspots^23^. FtsB and FtsL form a subcomplex in the absence of FtsQ via interactions between their transmembrane helices and membrane-proximal coiled coil regions^24^; they are also codependent regarding their stability and divisome localization^25^. The FtsQBL complex may have a structural role as a scaffold in the assembly of the divisome since FtsB and FtsL proteins are recruited to the divisome by FtsQ and the resulting FtsQBL complex then recruits late divisome components^25^.

Despite previous intensive studies on the FtsQBL complex, the atomic details of the interface between FtsQ and FtsB were not reported, and structural organization of periplasmic domains of the FtsQBL complex was not shown yet. In this study, we have determined the crystal structure of the periplasmic domain of FtsQ in complex with C-terminal fragment of FtsB, and also solved the solution structure of the periplasmic FtsQBL complex. Based on these structures, we have proposed a 2:2:2 model of the FtsQBL complex and provided insights into its organization in cell division.

## Results and Discussion

### Intermolecular interactions of FtsQ and FtsB

**Figure 1:**
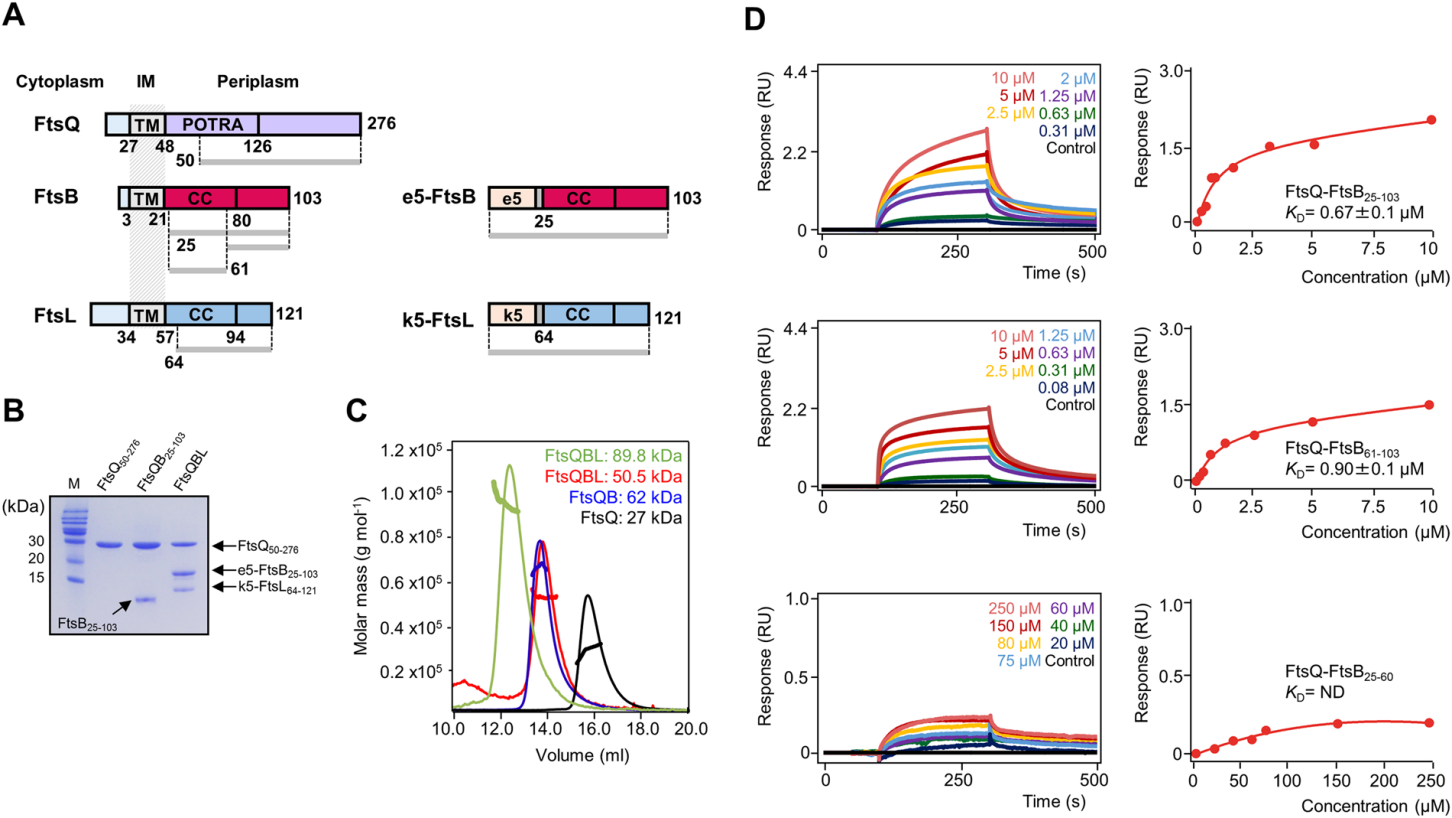
Mapping the FtsQ-FtsB-FtsL binding region. (**A**) Domain architecture of FtsQ, FtsB, and FtsL. Constructs are indicated by gray lines and reconstitution of FtsB and FtsL using e5- and k5-coils are shown. (**B**) SDS-PAGE of FtsQ_50–276_, FtsQ_50–276_/FtsB_25–103_, and FtsQ_50–276_/e5-FtsB_25–103_/k5-FtsL_64–121_ (FtsQBL) complexes. SDS-PAGE gels were visualized using Coomassie Blue. (**C**) FtsQBL heterohexamer (3 mg/mL, light green line), FtsQBL heterotrimer (3 mg/mL, red line), FtsQB (10 mg/mL, blue line), and FtsQ (10 mg/mL, black line) were analyzed by SEC-MALS. The thick line represents measured molecular mass. (**D**) Representative BLI assays of FtsQ_50–276_ with FtsB_25–103_, FtsB_61–103_, and FtsB_25–60_. BLI sensorgrams with different concentrations of analytes are indicated by different colors. Equilibrium analysis and calculated *K*_D_ are shown for each constructs at right panels. The experiments were repeated three times.

Previous studies showed that the periplasmic subcomplex of FtsB and FtsL can be reconstituted by replacing the membrane regions of both proteins with oppositely charged, soluble coiled coils (e5 and k5 in Fig. 1A)^20–22^. Using this technique, a stable binary complex (FtsBL) consisting of FtsB and FtsL was reconstituted by co-expressing e5-FtsB_25–103_ and k5-FtsL_64–121_ in *E. coli* cells (Fig. 1A and Supplementary Fig. S2). The ternary complex (FtsQBL) consisting of FtsQ, e5-FtsB, and k5-FtsL was reconstituted by mixing a wet cell pellet containing overexpressed FtsQ_50–276_ and a cell pellet containing overexpressed e5-FtsB_25–103_ and k5-FtsL_64–121_. FtsBL and FtsQBL complexes were co-eluted from an affinity column and co-migrated on size-exclusion columns (Fig. 1B,C). Stable FtsQ_50–276_-FtsB_25–103_ complex was formed (Fig. 1B), but FtsQ had no binding affinity with k5-FtsL_64–121_ in the absence of e5-FtsB_25–103_ (data not shown), consistent with previous study^20^. To precisely map the binding region between FtsQ and FtsB, two constructs (FtsB_25–60_ and FtsB_61–103_) with truncated N- or C-terminal regions of FtsB_25–103_ were designed and their interactions with FtsQ_50–276_ were examined in a bio-layer interferometry (BLI) experiment (Fig. 1D). FtsQ_50–276_ bound to the FtsB_25–103_ complex with a dissociation constant (*K*_D_) of 0.67 μM (Fig. 1D). Among the two FtsB constructs (FtsB_25–60_ and FtsB_61–103_), only FtsB_61–103_ binding to FtsQ_50–276_ was stable (*K*_D_ = 0.9 μM), suggesting that the C-terminal region of FtsB (primarily residues 61–103) mainly contributes to FtsQ binding (Fig. 1D), consistent with a previous result that the C-terminus of FtsB is necessary for interaction with FtsQ^25^. Although another previous study suggested that a membrane-proximal region of FtsQ is another interaction hotspot for FtsB binding^23^, stable binding of FtsQ_50–276_ with FtsB_25–60_ was not observed in our experiment (Fig. 1D).

### Crystal structure of the FtsQ-FtsB complex

**Figure 2:**
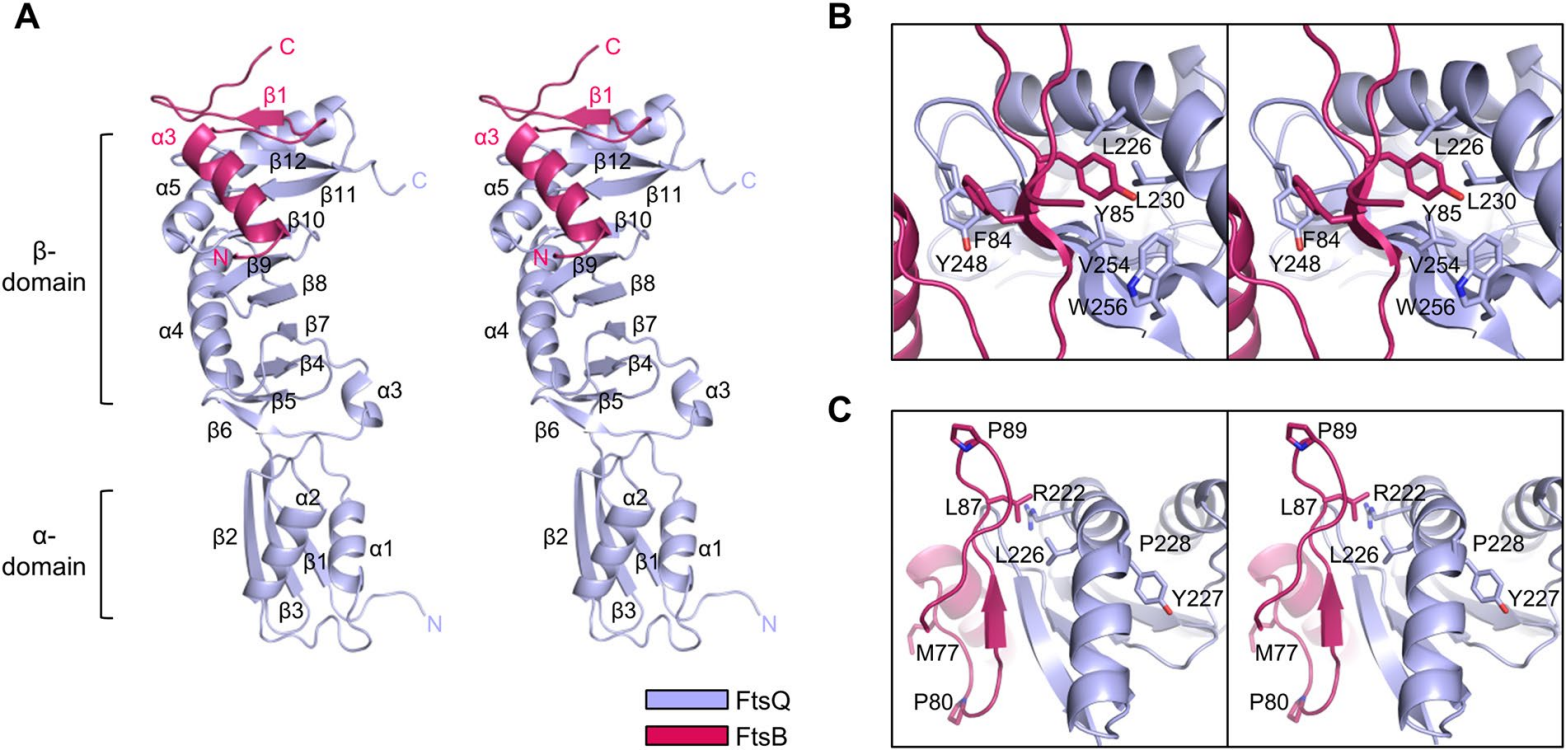
Overall structure of the FtsQ_50–276_/FtsB_25–103_ complex. (**A**) Stereo ribbon diagrams of the FtsQB complex. The electron density map of residues 61–95 was only observed for FtsB_25–103_. Each chain is shown in a different color. (**B**,**C**) Magnified stereo view showing details of the interaction at the interface of FtsQ and FtsB. Each chain is shown in a different color.

To gain further insights into structural organization of the FtsQB complex, we determined the 2.9-Å resolution structure of FtsQ_50–276_ bound to FtsB_25–103_ (Fig. 2, Supplementary Fig. S3, and Table 1). The electron density map of residues 25–60 was not observed for FtsB_25–103_, while nearly all sequences of FtsQ_50–276_ were found to be ordered with the exception of N- and C-terminal flexible regions (residues 50–52 and 262–276, respectively; Fig. 2A and Supplementary Fig. S3). Consistently, the electron density map of FtsB N-terminus (residues 22–63) was not observed for FtsB in the crystal structure of FtsQB complex, which was reported recently by other group^26^. One possibility regarding the lack of electron density is that the membrane-proximal helix is disordered in the crystal of FtsQB complex; however, the crystal structure of the 30 juxta-membrane amino acids of FtsB showed that it forms a canonical coiled coil^27^, thus we excluded this possibility. Another possibility is that the membrane-proximal helix is cleaved automatically during purification and crystallization, such that the remaining construct (FtsQ_50–276_-FtsB_61–103_) crystallizes easily. Given that previous molecular dynamics simulations of the FtsQBL complex showed that the central part of FtsB around the Leu60 residue presents with a helix-break^28^, we predicted that the central part of FtsB around Leu60 is prone to proteolysis.

**Table 1:** Data collection and refinement statistics.

	FtsQ_50–276_ & FtsB_25–103_
***A. crystal parameters***	
X-ray source	SPring-8 BL44XU
X-ray wavelength (Å)	1.0000
Space group	*P*2
Unit cell parameters	
*a, b, c* (Å)	63.6, 39.3, 73.2
*α, β, γ* (°)	90, 94.8, 90
Total/unique reflections	
Resolution range (Å)	50–2.92 (2.97–2.92)^a^
Total/unique reflections	33,095/8,124
Completeness (%)	100.0 (100.0)^a^
Average *I*/σ (*I*)	23.2 (2.2)^a^
*R*_merge_^b^ (%)	8.4 (98.0)^a^
***B. Model refinement statistics***	
Resolution range (Å)	50.0–2.97
*R*_work_/*R*_free_^c^ (%)	22.3/25.4
Number/average *B*-factor(Å^2^)	
Protein nonhydrogen atoms	1,964/89.1
Water oxygen atoms	21/84.7
Mg^2+^	2/69.8
R.m.s. deviations from ideal stereochemistry	
Bond lengths (Å)	0.012
Bond angles (°)	1.79
Protein-geometry analysis	
Ramachandran favored (%)	95.0
Ramachandran allowed (%)	4.2
Ramachandran outliers (%)	0.8

^a^Values in parentheses refer to the highest resolution shell.

^b^*R*_merge_ = Σ_hkl_Σ_i_ | *I*_i_(*hkl*) − < *I*(*hkl*) > |/Σ_*hkl*_Σ_i_ *I*_i_(*hkl*)_i_, where *I*(*hkl*) is the intensity of reflection *hkl*, Σ_hkl_ is the sum over all reflections, and Σ_i_ is the sum over i measurements of reflection *hkl*.

^c^*R* = Σ_hkl_ ||*F*_obs_| − |*F*_*c*alc_||/Σ_hkl_ |*F*_obs_|, where *R*_free_ was calculated for a randomly chosen 5% of reflections, which were not used for structure refinement and *R*_work_ was calculated for the remaining.

The overall structure of FtsQ_50–276_ consists of two domains, α and β, and the β-domain is responsible for FtsB_25–103_ binding (Fig. 2A). The solvent-accessible surface area buried at the interface between FtsQ and FtsB was 1330 Å^2^, approximately 20% of the monomer surface area of FtsQ. Furthermore, approximately 50% of the nonhydrogen atoms in the interface between FtsQ and FtsB are polar, as indicated by proximal isovelocity surface area calculations^29^. The FtsB sequence (residues 61–95) folds into one α-helix (α3) and one β-strand (β1; Fig. 2A). The α-helix-turn-β-strand motif begins with the α-helix (residues 64–75), followed by a kinked loop (residues 76–82), a short β-strand (residues 83–85), and another terminal kinked loop (residues 86–95; Fig. 2C); the two kinked loops contain two proline residues (Pro80 and Pro89; Fig. 2C). Interestingly, FtsQ α5 helix adopts a kinked shape by incorporating Pro228 in the middle and this shape appears to be crucial for its extensive contacts with FtsB. When Tyr precedes Pro in the amino acid sequence, it increases the tendency of the Pro residue to take on a *cis* conformation^30^; however, the Pro228 residue of FtsQ adopts a *trans* conformation (Fig. 2C).

### Binding modes of FtsQ in complex with FtsB

**Figure 3:**
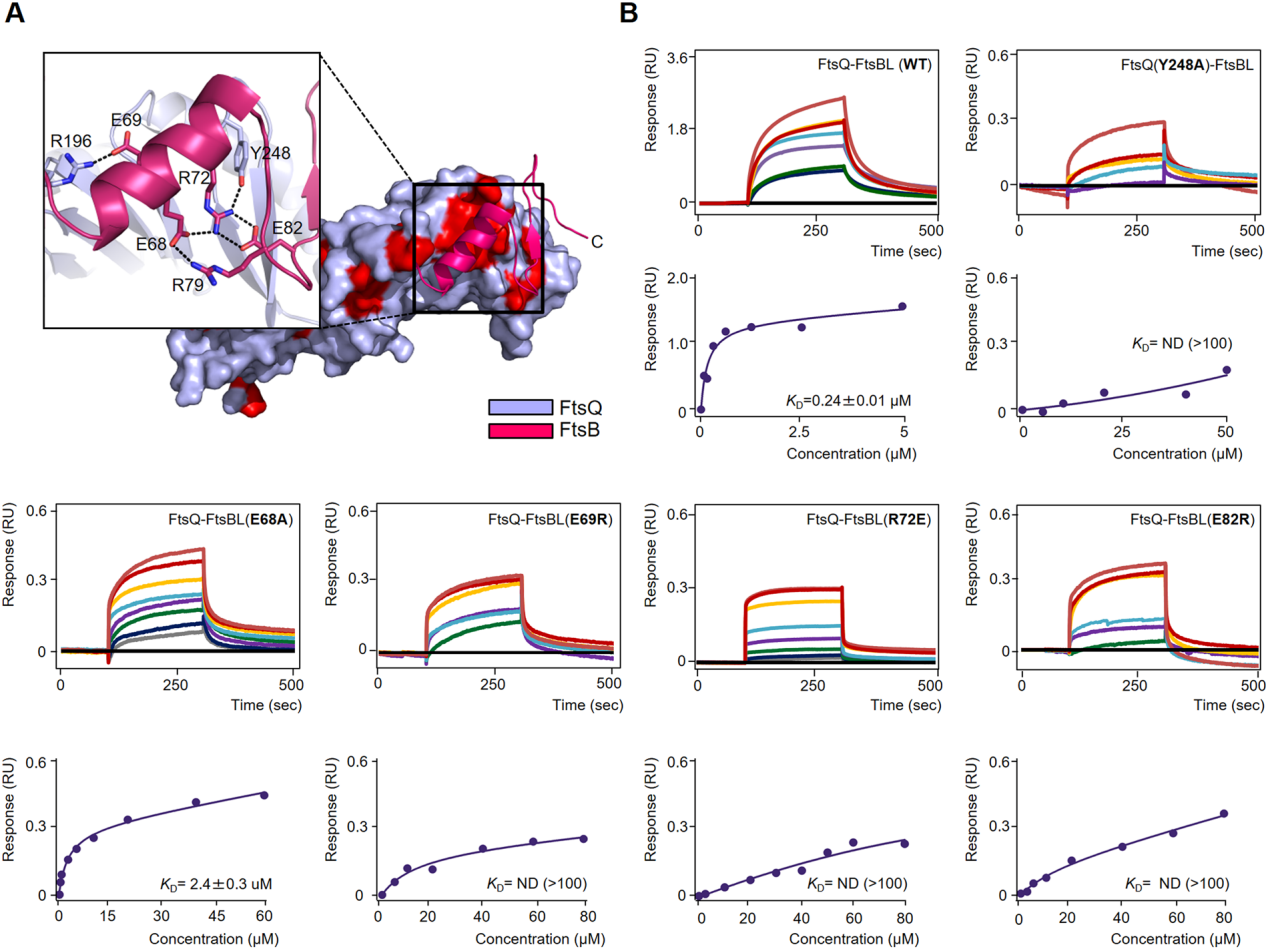
Molecular interactions of the FtsQB complex in detail. (**A**) Magnified views showing detailed interactions at the FtsQ and FtsB interfaces. Ribbon diagrams of FtsB and surface diagrams of FtsQ are shown. Red surfaces indicate 100% conserved residues of FtsQ (Supplementary Fig. S2). (**B**) Representative BLI assays of FtsQ-FtsBL complex WT and mutants (FtsQ Y248A, FtsB E68A, FtsB E69R, FtsB R72E, and FtsB E82R). BLI sensorgrams with different concentrations of analytes are indicated by different colors. Equilibrium analysis and calculated *K*_D_ are shown for each constructs at right panels.

Although previous mass spectrometric analysis by photo cross-linking indicated that the C-terminal regions of FtsQ and FtsB are crucial interaction hotspots, the detailed interactions proposed by the mass spectrometric analysis were completely different from those observed in our crystal structure (Fig. 2). Based on the mass spectrometric analysis, it was suggested that the C-terminus of FtsB around Met77 residue forms a β-sheet-like interaction with the C-terminal strand of FtsQ around Gly255 residue^23^. In contrast to these data, our crystal structure showed that the region around Met77 residue of FtsB did not form a secondary structure, and the direction of its side chain is opposite to the expected binding region of FtsQ (Fig. 2C). In our crystal structure, the C-terminal region of FtsB (residues 61–103) extensively interacts with the β-domain of FtsQ and mainly contributes to FtsQ binding. The kinked loop between the α-helix (residues 64–75) and β-strand (residues 83–85) wraps around the Tyr248 residue of FtsQ (Fig. 2B). To stabilize the kinked loop conformation, four FtsB residues (Glu68, Arg72, Arg79, and Glu82) form a cluster with extensive salt-bridge networks (Fig. 3A). FtsB Glu68 forms salt bridges with both Arg72 (2.8 Å) and Arg79 (2.8 Å), while Arg72 forms a salt bridge with Glu82 (2.8 Å and 3.0 Å, respectively; Fig. 3A); the Arg72 NH2 atom forms one additional interaction with the Glu68 OE2 atom (2.8 Å; Fig. 3A). The salt-bridge cluster of FtsB is crucial in hydrogen bond formation (2.7 Å) between the oxygen atom of FtsQ Tyr248 and the NH1 atom of FtsB Arg72 (Fig. 3A). Furthermore, FtsB Glu69 forms a salt bridge with FtsQ Arg196 (2.3 Å; Fig. 3A). Interestingly, the β-strand of FtsB makes a continuous β-sheet with that of FtsQ by anti-parallel stacking with β12 (Fig. 2C). FtsB Phe84 forms close contacts with FtsQ Tyr248, whereas Tyr85 resides in close proximity to the hydrophobic core of the FtsQ β-domain consisting of Leu226, Leu230, Val254, and Trp256 (Fig. 2B). FtsB Leu87 also forms extensive contacts with FtsQ Leu226 and the aliphatic portion of Arg222 (Fig. 2C).

To evaluate the contributions of interfacial residues of the FtsQB complex, the interfacial conserved residues of FtsQ (Tyr248) and FtsB (Glu68, Glu69, Arg72, and Glu82) were mutated and their interactions were examined by BLI. The binding affinities between FtsQ and FtsB were measured by immobilizing wild-type (WT) or mutants of GST-e5-FtsB in complex with k5-FtsL. As expected, mutation of the interfacial residues resulted in large reductions in affinity compared with WT (*K*_D_ = 0.24 μM). The *K*_D_ value of the Y248A mutant could not be measured due to weak binding, while E68A reduced affinity by approximately 10-fold (Fig. 3B). The charge reversal mutations E69R, R72E, and E82R of FtsB nearly abolished binding to FtsQ (Fig. 3B). This highlights the role of FtsQ Tyr248 and the FtsB α-helix-turn-β-strand motif as affinity determinants.

### FtsQ and FtsB interface *in vivo*

**Figure 4:**
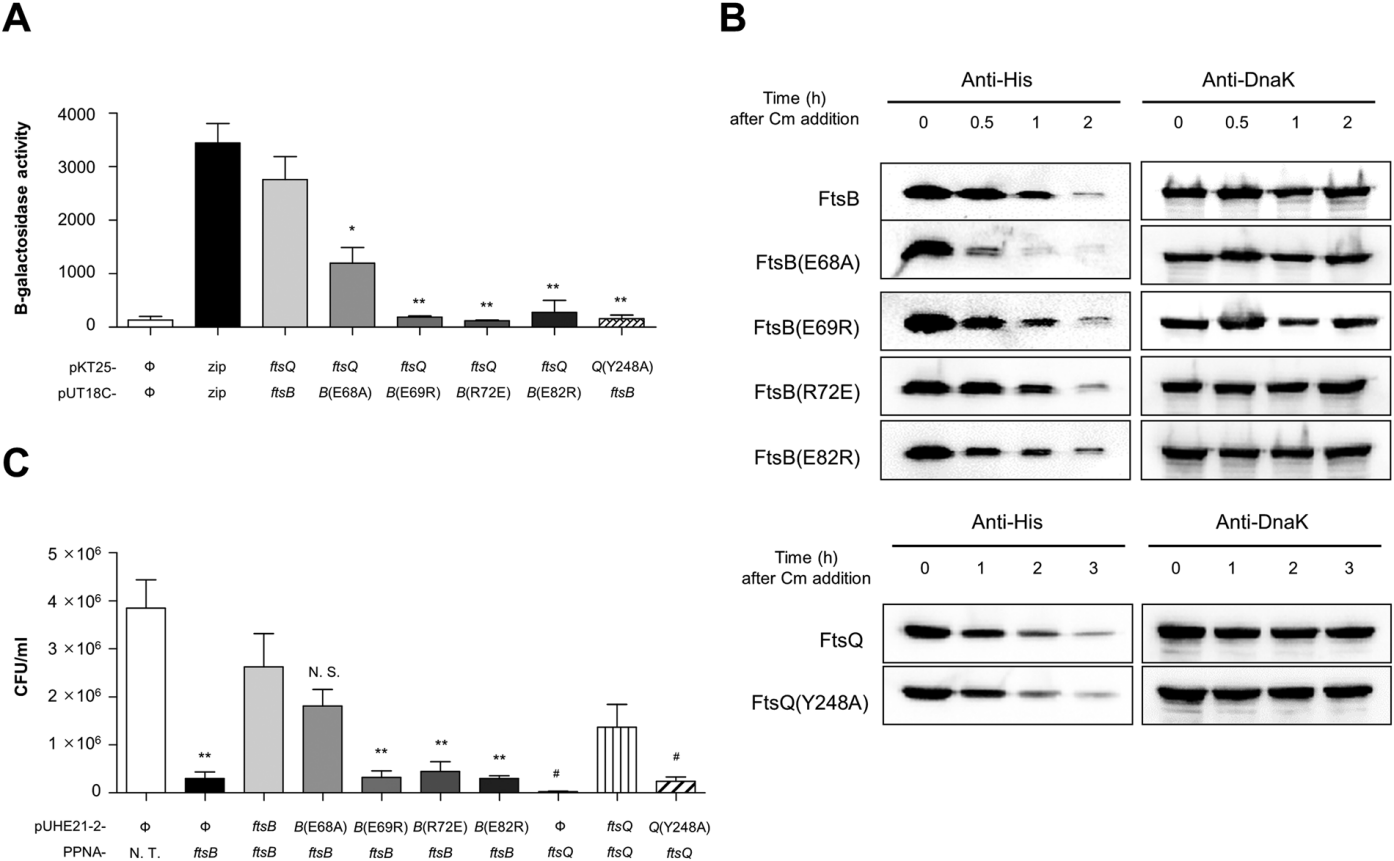
Molecular interactions of the FtsQB complex *in vivo*. (**A**) The effect of mutations at the interfacial residues of FtsQ and FtsB. The indicated plasmid pairs were introduced into a reporter strain *E. coli* BTH101. The interactions of FtsB and FtsQ were assessed by the bacterial two-hybrid system. The binding activities were measured by β-galactosidase assay and presented as Miller unit. Ф, backbone plasmid; *B*, ftsB; *Q*, ftsQ; *p < 0.05, **p < 0.005 compared with group expressing T18 fused WT FtsB and T25 fused FtsQ protein. (**B**) Comparison of the stability between WT and mutated proteins. *E. coli* MG1655 harboring plasmid pUHE21-2-*lacI*^*q*^ encoding C-terminal His-tagged WT or mutated protein was sampled at the indicated time points after adding chloramphenicol and subjected to the Western blotting. DnaK was used as a loading control. (**C**) The *in vivo* effect of point mutations on FtsQ and FtsB. The function of WT and mutated proteins was evaluated by peptide-conjugated PNA (PPNA). *E. coli* MG1655 strains harboring the indicated plasmids were treated with PPNA, complementary sequences in *E. coli* chromosome but not in the indicated plasmids. CFUs were measured after 3 h PNA treatment. Significant CFU differences are indicated as compared with control group expressing WT *ftsB* or *ftsQ*. *p < 0.05, **p < 0.005, N.S. not significant compared with *ftsB*; # *p* < 0.05 compared with *ftsQ*; N. T. not treated.

To verify the *in vitro* binding of the periplasmic regions of FtsB and FtsQ, we performed a bacterial two-hybrid assay based on a cyclic AMP signaling cascade^31^. As N-terminus of T18 or T25 fused Fts protein was toxic for *E. coli* and has a certain degree of instability problem, we constructed C-terminus-fused Fts proteins (T25-FtsQ, T18-FtsB)^18^. *E. coli* BTH101 reporter strain was co-transformed with a pair of indicated plasmids and the binding efficiency was determined by β-galactosidase assay (Fig. 4A). As shown in Fig. 4A, WT FtsB, and WT FtsQ interacted with each other. In similar with the BLI assay, FtsB (E68A) can bind to WT FtsQ, while the binding affinity was reduced approximately two-fold compared with WT FtsB. The mutated proteins except for the FtsB (E68A) diminished their ability to interact with WT FtsQ. It is consistent with *in vitro* binding results, indicating that the interfacial residues of FtsQ (Y248) and FtsB (E69, R72, and E82) play an important role in the formation of the FtsQB complex.

The interaction between FtsQ and FtsB is required for recruiting downstream Fts proteins. When FtsQ or FtsB is depleted or introduced with deleterious mutation, it leads to filamentation and eventually cells death. To evaluate the *in vivo* function of FtsQB interfacial residues, we conducted a viability assay of cells expressing WT or mutated proteins with peptide nucleic acids (PNAs). A previous study showed that peptide-conjugated PNAs (PPNAs) targeting essential genes had bactericidal effects on *E. coli* cells^32–34^. Ribosome binding site is known to be a key PNA target site for inhibiting translation^32^. As the tight binding of PNA to mRNA interferes with ribosome function, protein translation is prevented^33^. Based on these principles, we designed four antisense PPNAs that are complementary to the translation initiation sites of *ftsB* and *ftsQ* (Supplementary Fig. S4). The growth of the *E. coli* MG1655 strain was measured to examine the inhibitory effects of antisense PPNA1, PPNA2, PPNA3, and PPNA4. *ftsB*-targeting PPNA1 and *ftsQ*-targeting PPNA3 strongly inhibited bacterial growth and filamentation of *E. coli* was observed (Supplementary Figs S4 and S5). Thus, we utilized PPNA1 and PPNA3 for further experiments. We constructed inducible plasmids expressing WT FtsB, WT FtsQ, FtsB mutants (E68A, E69R, R72E, and E82R), and FtsQ mutant (Y248A). As the used PPNAs have no complementary sequences to the recombinant plasmids, they do not interfere with the protein expression in plasmids. Strains expressing WT or mutated proteins showed no significant differences in colony-forming units (CFU) at 3 h without PPNA. Cells expressing the WT proteins (FtsB or FtsQ) in the plasmid recovered bacterial growth when treated with PPNA, but the cell expressing the mutated protein did not recover growth except for the FtsB (E68A) (Fig. 4C). Also, filamentation was observed in bacteria expressing mutated proteins lacking the ability to recover growth when treated with PPNA (Supplementary Fig. S5). To exclude a possibility that the protein stability could affect the function of the mutated protein, we also determined *in vivo* protein stability by blocking *de novo* protein synthesis. The plasmid encoding FtsB or FtsQ fused with His tag at the C-terminus was introduced into *E. coli* MG1655, and the stability of the mutated protein was compared with WT FtsB or FtsQ (Fig. 4B and Supplementary Fig. S6). As a result, the mutated proteins except for FtsB (E68A) showed similar results as WT. Although FtsB (E68A) was unstable compared with WT FtsB, this mutated protein FtsB (E68A) can complement the function of WT FtsB and interact with FtsQ. Taken together, it suggests that the mutations of interfacial conserved residues of FtsQ (Y248) and FtsB (E69, R72, and E82) do not affect the protein stability but abolish the interaction between FtsB and FtsQ.

### Oligomeric states of FtsQB and FtsQBL in solution

Previously, contradictory results were reported for the oligomeric state of the FtsQ protein. Dimerization of FtsQ via its transmembrane region (or C-terminal 30 residues) was observed in a bacterial two-hybrid experiment^18,35^. In contrast, a direct experiment for determining the oligomeric state of FtsQ in solution was performed using analytical ultracentrifugation indicating that FtsQ is a monomer^19^. However, we cannot exclude the possibility that the transmembrane region of FtsQ affects the oligomerization state of FtsQ, since the analytical ultracentrifugation experiment was performed using the periplasmic region of FtsQ^19^. Even though we also used the periplasmic regions of FtsQ, FtsQB, and FtsQBL, we hypothesized that their oligomeric states in solution depends on the concentration and that further oligomerization may occur at high concentrations. To analyze the concentration-dependent oligomeric states of FtsQ, FtsQB, or FtsQBL in solution, analytical gel filtration was performed using a Superdex 200 (10/300 GL) column and the experimental Stokes radii (R_H_) were compared with those calculated from structure models (Supplementary Fig. S7). Structure models of FtsQ, FtsQB, and FtsQBL for calculating R_H_ values were generated based on our crystal structure of FtsQB and SAXS structure of FtsQBL as described below. The invisible segments of the N- and C-termini of FtsQ, FtsB, and FtsL were modeled as flexible loops (Supplementary Fig. S7).

**Figure 5:**
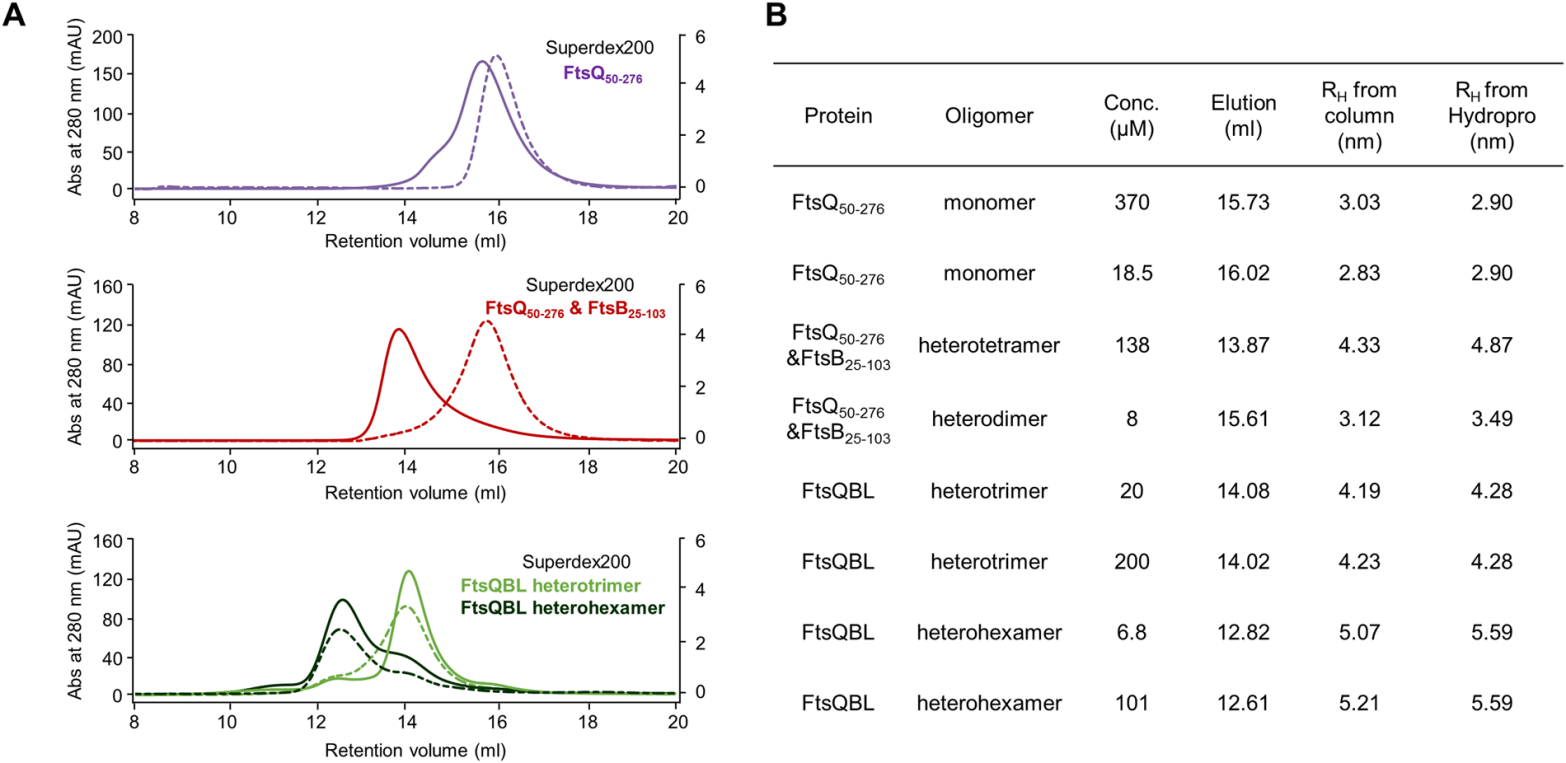
Concentration-dependent oligomerization of FtsQ_50–276_/FtsB_25–103_. (**A**) Analytical gel filtration profiles of FtsQ_50–276_ (purple), FtsQ_50–276_/FtsB_25–103_ (red), FtsQBL heterotrimer (light green), and FtsQBL heterohexamer (dark green) at high (101–370 μM; solid lines and left y-axis) and low (6.8–20 μM; dotted lines and right y-axis) concentrations. (**B**) Summary of the hydrodynamic analysis in Fig. 5A. R_H_ values from column were compared to those calculated from structure models (Supplementary Fig. S7) using HYDROPRO program.

For high and low concentrations of FtsQ_50–276_ (370 and 18.5 μM, respectively), the apparent R_H_ (3.03 and 2.83 nm, respectively) was closer to the theoretical R_H_ of FtsQ_50–276_ monomer (2.90 nm), suggesting that FtsQ mainly exists as a monomer in solution (Fig. 5). In contrast, for the FtsQB complex, the apparent R_H_ (4.33 nm) was closer to that of the 2:2 FtsQB heterotetramer (4.87 nm) at high concentrations (138 μM), whereas the apparent R_H_ (3.12 nm) was closer to that of the 1:1 FtsQB heterodimer (3.49 nm) at low concentrations (8 μM; Fig. 5). To further support the 2:2 FtsQB heterotetramer model, the molecular weight of FtsQB at high concentrations (138 μM) was measured by size-exclusion chromatography with multi-angle light scattering (SEC-MALS). Indeed, the molecular mass of FtsQB was consistent with a 2:2 complex of FtsQ and FtsB (Fig. 1C). These results suggest that FtsB mediates dimerization of FtsQB since FtsQ exists as a monomer at high and low concentrations. For the FtsQBL complex, heterohexameric and heterotrimeric forms were eluted separately at the gel filtration during purification and they were collected separately. Each form was stable as indicated by SEC-MALS (Fig. 1C), and further used for the analysis of analytical gel filtration. At high and low concentrations of the FtsQBL complex (6.8–20 μM and 101–200 μM, respectively), the apparent R_H_ values of heterohexameric FtsQBL were closer to that of the 2:2:2 FtsQBL model (5.59 nm), while the R_H_ values of heterotrimeric FtsQBL were closer to that of the 1:1:1 FtsQBL model (4.28 nm) (Fig. 5). This suggests that both heterohexameric and heterotrimeric forms of FtsQBL exist in a stable form regardless of its concentrations.

### Structural organization of the FtsQB tetramer

We next examined structural organization of the FtsQB heterotetramer in solution. When we evaluated crystal packing of the FtsQB complex, a potential FtsQB heterotetramer was generated through crystallographic symmetry operations and exhibited an elongated structure with overall dimensions of 30 × 40 × 150 Å, such that the FtsQB heterodimer dimerized in an antiparallel head-to-head fashion via the N-terminal α-domains of FtsQ subunits (Supplementary Fig. S7C). Although we speculated that FtsQB forms a larger oligomer via FtsB based on analytical gel filtration data, we could not rule out the possibility that the FtsQB heterotetramer in solution was formed by the same interface observed in the crystal contact. Thus, we examined if the crystallographic interface is involved in forming the FtsQB heterotetramer. At the dimer interface of FtsQ, strictly conserved Leu57 and Phe87 residues form a hydrophobic core at the interface (Supplementary Fig. S2A); therefore, we hypothesized that the FtsQB L57R or F87A mutant exists as a heterodimer regardless of its concentration if Leu57 and Phe87 are crucial residues for the assembly of the FtsQB heterotetramer. However, both L57R and F87A mutants assembled into a 2:2 heterotetramer only at high concentrations (Supplementary Fig. S8), suggesting that the observed FtsQB heterotetramer in solution was not formed via the N-terminal α-domains of FtsQ subunits.

We next hypothesized that the coiled coil region of FtsB is responsible for the dimerization of FtsQB heterodimers. In this case, we predicted that a helix-breaking L46P mutant of FtsQB inhibits dimerization of FtsQB heterodimers. Indeed, some portions of FtsQB L46P mutant assemble into 1:1 heterodimers, even at high concentrations (Supplementary Fig. S8). These results suggest that the coiled coil region of FtsB is crucial for the assembly of the FtsQB heterotetramer in solution. In the 2:2 FtsQB heterotetramer model, the N-terminal directions of both FtsB subunits are oriented in the same direction, and thus the transmembrane region of FtsB may also dimerize to facilitate homodimerization of full-length FtsB. This may explain why higher-order oligomerization of FtsQB was observed only at high concentrations. Consistent with our findings, a previous study reported that FtsB self-associates via periplasmic coiled coil and transmembrane regions^26^. For high and low concentrations of e5-FtsB_25–103_/k5-FtsL_64–121_ (191 and 22.2 μM, respectively), the apparent R_H_ (both 4.50 nm) was closer to the theoretical R_H_ of the 2:2 heterotetramer of e5-FtsB_25–103_/k5-FtsL_64–121_ (4.38 nm), suggesting that e5-FtsB_25–103_/k5-FtsL_64–121_ mainly exists as a heterotetramer in solution (Supplementary Fig. S8). In agreement with this, a recent study using FRET analysis and computational methods also showed that the FtsBL complex forms a tetramer^36^.

### SAXS structure of FtsQBL complex

**Figure 6:**
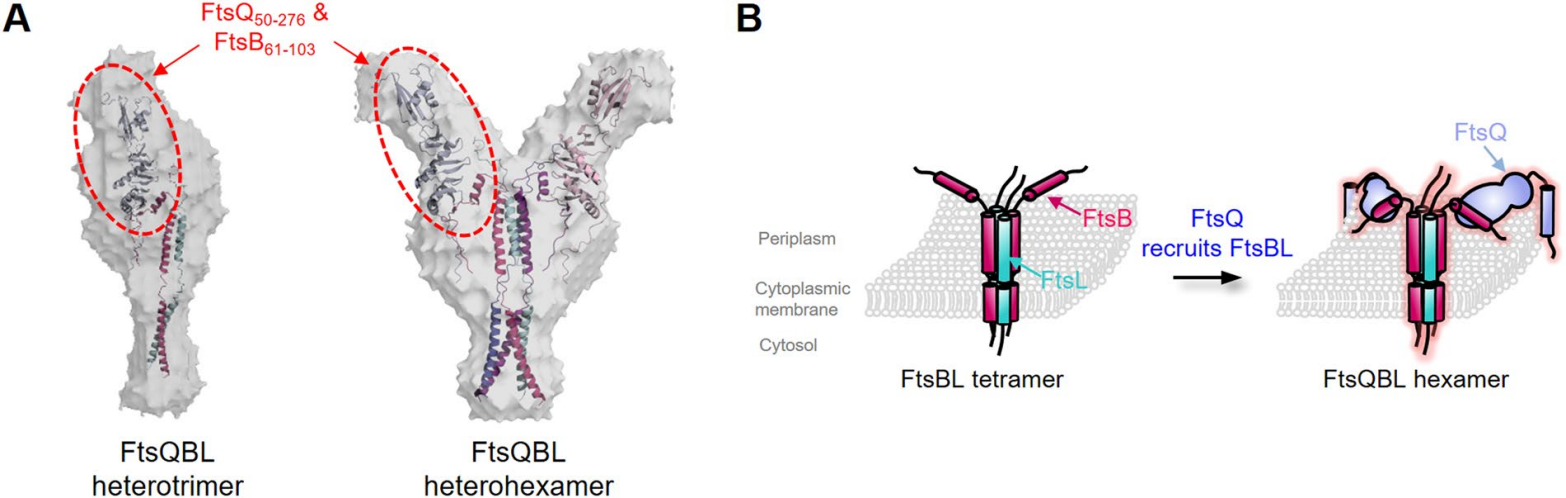
SAXS solution structure of FtsQBL and overall model of the FtsQBL complex. (**A**) SAXS solution structures of FtsQBL 1:1:1 trimer and 2:2:2 hexamer. Structural models of the FtsQBL complexes (1:1:1 and 2:2:2) were reconstructed by using the *ab initio* shape determination program DAMMIF. For each protein, 10 independent models were generated, compared, and an averaged model was calculated using the program DAMAVER. Surface rendering of the structural models was achieved using the program PyMOL. To compare overall shapes and dimensions, the ribbon diagram of the atomic models of FtsQBL proteins were superimposed onto the reconstructed dummy atoms models using the program SUPCOMB. NSD = 7.039 for the 1:1:1 model and 3.800 for the 2:2:2 model. (**B**) Overall model of Y-shaped FtsQBL. The FtsQBL association by recruiting the FtsBL complex to FtsQ is shown. Each chain is represented in a different color.

To determine the overall conformation of the FtsQBL complex in solution, SAXS data were collected. The 1:1:1 or 2:2:2 FtsQBL complex was used to determine the *ab initio* structure of the molecular envelope at low and high concentrations, respectively (Fig. 6A and Supplementary Fig. S9). Interestingly, the overall envelope of the FtsQBL 2:2:2 complex showed an elongated “Y” shape (Fig. 6A). Even though the envelope did not show sufficient details to position the individual domains of FtsQ, FtsB, and FtsL, we observed that the large envelope with a concave surface fit reasonably well with the shape of FtsQ (Fig. 6A). To incorporate the two FtsQ molecules into the large envelope with a concave surface, the N-terminal directions of FtsQ and FtsBL should be opposite. This was unexpected as the previous models formed by other groups possessed N-terminal transmembrane helices oriented in the same direction^20^. The crystal structure of the FtsQ_50–276_/FtsB_25–103_ complex was successfully incorporated into the molecular envelope (Fig. 6A and Supplementary Fig. S9), indicating that the overall conformation of the FtsQ_50–276_-FtsB_25–103_ complex is quite rigid in solution. The additional envelope was assigned to the positions of the heterotetrameric coiled coil and FtsBL e5- and k5-coils (Fig. 6A). It was recently suggested that the FtsBL subcomplex is a 2:2 heterotetramer with an uninterrupted FtsL helix linking the transmembrane and periplasmic coiled coil^36^; therefore, the Y-shaped FtsQBL may fit well into the curved membrane for membrane anchoring (Fig. 6B). Due to the low resolution of the SAXS envelope, it was difficult to clearly define the structural assembly of FtsQBL; therefore, further structural determination is required. Although we observed 2:2 FtsQB heterotetramer and 2:2:2 FtsQBL heterohexamer formation *in vitro*, whether these forms exist *in vivo* remains unclear and further studies are required to reveal the physiological stoichiometry of the divisome. However, it should be noted that FtsQBL was previously suggested to form a 2:2:2 complex at the divisome^37^. Although additional studies are needed to fully understand structural organization of FtsQBL in divisome assembly, our data provide a foundation for further investigation.

## Conclusion

Divisome proteins are essential for bacterial viability and its assembly and disassembly of the divisome occurs for each round of cell division in a predominantly hierarchical order^3^. Among the divisome proteins the FtsQBL complex was suggested to be a scaffold protein in the assembly of the divisome^25^. The FtsQBL complex is essential for bacterial viability^4^; thus, exploiting it would be useful in discovering antibiotics that curb bacterial growth. Despite previous biochemical studies on the FtsQBL complex, the atomic details of crucial interaction hotspots between FtsQ and FtsB were not reported. In this study, we have determined the crystal structure of the periplasmic domain of FtsQ in complex with C-terminal fragment of FtsB, showing that the C-terminal region of FtsB is a key binding region of FtsQ. The identification of crucial residues in protein-protein interactions between FtsQ and FtsB would be useful not only to provide insights into mechanisms of recognition between them, but also to indicate the regions to be targeted with inhibitors. We also solved the solution structure of the periplasmic FtsQBL complex by SAXS analysis. It should be noted that the roles of transmembrane helices of the FtsQBL complex should be explored in future studies because the periplasmic subcomplex of FtsQBL without the N-terminal membrane regions of FtsQ, FtsB, and FtsL was reconstituted *in vitro* in this study. However, we could propose a 2:2:2 Y-shaped model of the FtsQBL complex based on our SAXS structure, providing insights into its organization on the curved membrane for membrane anchoring.

## Methods

### Protein expression and purification

Constructs comprising residues 50–276 of FtsQ, residues 25–103 of FtsB, and residues 64–121 of FtsL were cloned into the pET21a (Novagen, Madison, WI, USA), pGST2^38^, and pRSF-duet (Novagen) vectors, respectively. To prepare FtsB-FtsL complexes, the sequence encoding the e5-coil and k5-coil was inserted into the N-terminus of FtsB_25–103_ and FtsL_64–121_, respectively^20,22^. The pET21a, pGST2, and pRSF-duet vectors contained a C-terminal six-histidine tag, an N-terminal GST tag, and an N-terminal six-histidine tag, respectively (Supplementary Table S1).

FtsQ_50–276_ was expressed in *E. coli* BL21 (DE3) cells induced with 0.5 mM isopropyl-β-D-1-thiogalactopyranoside (IPTG). For cell lysis, cell pellets were resuspended in buffer A (20 mM Tris-HCl pH 7.9, 500 mM NaCl, and 5 mM imidazole) containing 1 mM phenylmethylsulfonyl fluoride. Cells were lysed with a microfluidizer (Microfluidics, Westwood, MA, USA) and then lysed cells were centrifuged at 4600 × *g* (Vision V506CA rotor) for 30 min at 277 K to pellet the cell debris; the supernatant was applied to a Ni-affinity column (GE Healthcare, Little Chalfont, UK) pre-equilibrated with buffer A. The eluate was further purified by gel filtration on a HiLoad 16/60 Superdex 75 column (GE Healthcare) that had been pre-equilibrated with buffer B (20 mM Tris-HCl pH 8.0 and 200 mM NaCl).

FtsB_25–103_ was expressed in *E. coli* BL21 (DE3) cells induced with 0.5 mM IPTG. The e5-FtsB_25–103_/k5-FtsL_64–121_ complex was co-expressed in *E. coli* BL21 (DE3) cells induced with 0.5 mM IPTG. For the FtsQB complex, cell pellets of FtsQ_50–276_ and FtsB_25–103_ were resuspended together in buffer B and mixed in a 1:1 ratio. Cells were lysed with a microfluidizer and the lysed cells were centrifuged at 4600 × g for 30 min at 277 K to pellet the cell debris; the supernatant was then applied to a glutathione-Sepharose column (GE Healthcare) pre-equilibrated with buffer B. Proteins were eluted with buffer B containing 15 mM reduced glutathione. The eluates were desalted into buffer B, cleaved by TEV protease, and re-passed through a glutathione-Sepharose column pre-equilibrated with buffer B to remove the GST tag. The eluates were further purified by gel filtration on a HiLoad 16/60 Superdex 200 column (GE Healthcare) that had been pre-equilibrated with buffer B. For the FtsQBL complex, cell pellets of FtsQ_50–276_ and the e5-FtsB_25–103_/k5-FtsL_64–121_ complex were resuspended together in buffer A and mixed in a 1:1 ratio. The FtsQBL complex was purified using the same method of purifying the FtsQB complex, except the supernatant was applied to a Ni-affinity column pre-equilibrated with buffer A before using the glutathione-Sepharose column. Interestingly, two major peaks were eluted separately after size-exclusion chromatography, which corresponds to the 2:2:2 and 1:1:1 complexes of FtsQBL, respectively.

For BLI measurements, constructs of FtsB_25–103_ with the N-terminal e5-coil (WT, E68A, E69R, R72E, and E82R), FtsB_25–60_, and FtsB_61–103_ were cloned into the pGST2 vector, while the FtsQ_50–276_ Y248A construct was cloned into the pET21a vector. For BLI experiments, each e5-FtsB_25–103_ protein (WT, E68A, E69R, R72E, and E82R) was co-expressed with k5-FtsL_64–121_ in BL21 (DE3) cells, while FtsQ_50–276_ (WT and Y248A), FtsB_25–60_ and FtsB_61–103_ were also expressed in BL21 (DE3) cells. All proteins were purified using a glutathione-Sepharose or a Ni-affinity column, followed by gel filtration on a HiLoad 16/60 Superdex 200 column that had been pre-equilibrated with buffer B. The primers used to construct bacterial strains and plasmids can be found in Supplementary Table S2.

### Crystallization and data collection

Crystals of the FtsQ_50–276_/FtsB_25–103_ complex were grown by the sitting-drop vapor diffusion method by mixing equal volumes (0.8 μL) of each protein solution (~10 mg/mL in buffer containing 20 mM Tris-HCl pH 8.0 and 200 mM NaCl) and reservoir solutions. A reservoir solution consisting of 15% PEG6000, 0.2 M magnesium chloride, and 0.1 M Tris-HCl pH 8.0, was used to grow crystals of the FtsQ_50–276_/FtsB_25–103_ complex. Crystals of the FtsQ_50–276_/FtsB_25–103_ complex reached maximum size within 1–2 days at 296 K. The crystals were soaked in 15% PEG6000, 0.2 M magnesium chloride, 0.1 M Tris-HCl pH 8.0, and 25% glycerol before being flash-frozen in a nitrogen stream at 100 K. Native data for the FtsQ_50–276_/FtsB_25–103_ complex were collected at the beamline BL44XU at SPring-8, Japan. The raw data were processed and scaled using the program suite HKL2000^39^. Table 1 summarizes data collection statistics.

### Structure determination and refinement

The structure of the FtsQ_50–276_/FtsB_25–103_ complex was solved by the molecular replacement method using the monomer model (chain A) of FtsQ from *E. coli* (PDB ID: 2VH1)^19^. A cross-rotational search followed by a translational search was performed using the Phaser program^40^. Subsequent manual model building was carried out using the COOT program^40^ and restrained refinement was performed using the REFMAC5 program^41^. Several rounds of model building, simulated annealing, positional refinement, and individual *B*-factor refinement were performed. Table 1 lists the refinement statistics. The crystallographic asymmetric unit of the FtsQ_50–276_/FtsB_25–103_ complex contained one heterodimer. The refined model included 21 water molecules and 100% of the residues were in the most allowed region of the Ramachandran plot. No electron density was observed for residues 25–60 in FtsB.

### Size exclusion chromatography with multi-angle light scattering

SEC-MALS experiments were performed using an FPLC system (GE Healthcare) connected to a Wyatt MiniDAWN TREOS MALS instrument and Wyatt Optilab rEX differential refractometer (Santa Barbara, CA, USA). A Superdex 200 10/300 GL (GE Healthcare) gel-filtration column pre-equilibrated with buffer B was normalized using ovalbumin protein. Proteins were injected (3–10 mg/mL) at a flow rate of 0.4 mL/min. Data were analyzed using the Zimm model for fitting static light-scattering data and graphed using EASI graph with a UV peak in ASTRA V software (Wyatt).

### Analytical gel filtration

Purified FtsQ_50–276_, FtsQ_50–276_-FtsB_25–103_ complex, FtsQ_50–276_-FtsB_25–103_ mutants (L57R and F87A), e5-FtsB_25–103_/k5-FtsL_64–121_ complex, and FtsQ_50–276_/e5-FtsB_25–103_/k5-FtsL_64–121_ complex proteins were subjected to analytical gel filtration chromatography on a Superdex 200 (10/300 GL) column with buffer B at a constant flow rate of 0.5 mL/min. A standard curve was obtained using molecular weight markers (Sigma-Aldrich, St. Louis, MO, USA). The Stokes radii of β-amylase, alcohol dehydrogenase, carbonic anhydrase, and cytochrome *c* were calculated from the crystal structures of each protein (PDB codes: 1FA2, 2HCY, 1V9E, and 1HRC, respectively) using the HYDROPRO program^42^.

### BLI measurements

BLI measurements were performed to evaluate binding between FtsQ_50–276_ and the three constructs of GST-fused FtsB proteins (FtsB_25–60_, FtsB_61–103_, and FtsB_25–103_) using a BLItz system (ForteBio, Menlo Park, CA, USA). GST-fused FtsB proteins (FtsB_25–60_, FtsB_61–103_, and FtsB_25–103_) were immobilized on an AR2G biosensor chip surface using amine coupling in 20 mM HEPES (pH 7.5). The surface was activated by 5-min immersion in NHS/EDC, followed by a 5-min immersion of the GST-fused FtsB proteins in 10 mM sodium acetate (pH 4.0). Subsequently, the AR2G biosensor chip was blocked by 5-min immersion in 1 M ethanolamine (pH 8.9) and then the surface was equilibrated with running buffer (20 mM Tris-HCl pH 8.0 and 200 mM NaCl). To determine if interactions occur between the GST-fused e5-FtsB_25–103_/k5-FtsL_64–121_ complex (WT, E68A, E69R, R72E, and E82R) and FtsQ_50–276_ (WT and Y248A), the prepared AR2G biosensor chip was dipped in 0.1–80 μM protein solution in running buffer. Both associations and dissociations were measured for 200 s. Data points were collected at 100 s after the association of analyte started and dissociation constant (*K*_D_) calculation was done using GraphPad Prism program (GraphPad).

### Bacterial two-hybrid assay

The BACTH (Bacterial Adenylate Cyclase Two-Hybrid) system based on recovery of adenylate cyclase (CyaA) activity through heterodimerization of recombinant fused proteins was performed in *E. coli* BTH101 reporter strains (*cya* mutant)^31^. The reporter strain *E. coli* BTH101 was co-transformed with the fusion plasmid pair and cultured in LB broth in the presence of IPTG (0.5 mM) at 30 °C for 18 h. To determine the efficiencies of interactions between proteins, β-galactosidase activity was measured.

### Plasmid construction

We employed an isothermal assembly with DNA fragments, including linearized plasmid and PCR products. Preparation of fragments for isothermal assembly was performed by PCR and target sequences were amplified to overlap the adjacent fragment (~30 base pairs). The prepared fragments were assembled by incubation at 50 °C for 1 h in reaction buffer (25% PEG8000, 500 mM Tris-HCl pH 7.5, 50 mM DTT, 1 mM of dNTPs, and 5 mM NAD) with several enzymes (T5 exonuclease, Phusion polymerase, and Tag DNA ligase).

### Western blot analysis

Cells were sampled at the indicated time points and harvested. Pellets were immediately frozen in liquid nitrogen and stored at −20 °C until further analysis. Next, 1× sample buffer was added to the frozen pellet and sonicated. Whole-cell fractions were separated by molecular weight using SDS-PAGE, followed by transfer to a polyvinylidene difluoride membrane. The membrane was blocked with 5% non-fat dry milk and probed with an anti-Histidine antibody (1: 3,000 dilutions, Sigma, St. Louis, MO, USA), anti-DnaK antibody (1: 10,000 dilutions, Biolegend, San Diego, CA, USA) as primary antibodies. Anti-mouse IgG conjugated to peroxidase (1: 5,000 dilutions, Gendepot, Katy, TX, USA) was used as secondary antibodies in all western blot experiments. The chemiluminescent signals were developed with ECL reagent (Promega, Fitchburg, WI, USA) and detected by ChemiDoc (Bio-Rad, Hercules, CA, USA).

### Analysis of protein stability

*E. coli* strain encoding FtsB-His or FtsQ-His protein was grown aerobically at 37 °C for 3 h in LB medium supplemented with 0.1 mM IPTG. Subsequently, the culture was harvested and resuspended the cell pellet in fresh LB medium containing chloramphenicol (0.2 mg/mL) to block *de novo* protein synthesis. After adding chloramphenicol, the cells were subjected to western blot analysis at the indicated time points.

### PNA knockdown experiment

PNAs used in this study were commercially synthesized by Panagene (Daejeon, Korea). All PNAs were covalently conjugated to the cell-penetrating peptide (KFF)_3_K (in which K is lysine and F is phenylalanine) at the carbon terminus with an O linker. To determine the bacterial susceptibility to PNA, bacteria harboring pUHE21–2-*lacI*^*q*^ encoding WT or mutated proteins were grown aerobically at 37 °C for 3 h in LB medium supplemented with 0.1 mM IPTG. Subsequently, the cultures were diluted to 5 × 10^4^ CFU/mL and incubated with 12.5 μM anti-ftsB PPNA1 or anti-FtsQ PPNA3 at 37 °C. After incubation for 3 h, the CFUs were determined on LB agar plates.

### Solution SAXS measurements

SAXS measurements were carried out using the 4C SAXS II beamline of Pohang Light Source II with 3 GeV power at the Pohang University of Science and Technology (POSTECH), Korea. Light source from the In-vacuum Undulator 20 (IVU20: 1.4 m length, 20 mm period) of the Pohang Light Source II storage ring was focused with a vertical focusing toroidal mirror coated with rhodium and monochromatized with a Si (111) double crystal monochromator, yielding an X-ray beam wavelength of 0.734 Å. The X-ray beam size at the sample stage was 0.1 (V) × 0.3 (H) mm^2^. A two-dimensional charge-coupled detector (Mar USA, Inc., Evanston, IL, USA) was employed. Sample-to-detector distances of 4.00 and 1.00 m were used for SAXS. The magnitude of the scattering vector, *q* = (4*π*/*λ*) sin *θ*, was 0.09 nm^−1^ < *q* < 6.00 nm^−1^, where 2*θ* is the scattering angle and *λ* is the wavelength of the X-ray beam source. The scattering angle was calibrated with a polystyrene-b-polyethylene-b-polybutadiene-b-polystyrene block copolymer standard. We used a quartz capillary with an outside diameter of 1.5 mm and wall thickness of 0.01 mm as the solution sample cell. All scattering measurements were carried out at 277 K using an FP50-HL refrigerated circulator (JULABO, Seelbach, Germany). The SAXS data were collected in ten successive frames of 3 s each to monitor radiation damage. Measurements of FtsQBL protein solutions were carried out over a small concentration range of 1.0–3.0 mg/mL. Each 2D SAXS pattern was radially averaged from the beam center and normalized to the transmitted X-ray beam intensity, which was monitored with a scintillation counter placed behind the sample. Scattering of specific buffer solutions was used as experimental background. Structural parameters obtained from the SAXS data are shown in Supplementary Table S3. The R_g_ (radius of gyration) values were estimated from the scattering data by Guinier analysis^43^. The pair distance distribution p(r) function was obtained through the indirect Fourier transform method using the program GNOM^44^.

### Construction of 3D structural models

To reconstruct molecular shapes, the *ab initio* shape determination program DAMMIF^45^ was used. For each model reconstruction, ten independent models were selected and averaged aligned using the program DAMAVER^46^. SAXS curves were calculated from the atomic models using the program CRYSOL^47^. To compare overall shapes and dimensions, ribbon diagrams of the atomic crystal models were superimposed onto the reconstructed dummy atom models using the program SUPCOMB^48^.

Atomic coordinates and structure factors for the FtsQ_50-276_/FtsB_25-103_complex have been deposited in the Protein Data Bank (PDB ID code 5Z2W).

## Electronic supplementary material

Supplementary figures and tables
