# Supplementary material for: Structural Insights into the FtsQ/FtsB/FtsL Complex, a Key Component of the Divisome
Source: Sci Rep. 2018 Dec 24;8:18061. doi: 10.1038/s41598-018-36001-2 (PMC6305486; doi:10.1038/s41598-018-36001-2)
Supplement: Supplementary file 1 — Supplementary figures and tables [file 41598_2018_36001_MOESM1_ESM.docx]

**Structural Insights into the FtsQ/FtsB/FtsL Complex, a Key Component of the Divisome**

Yuri Choi^1,*^, Jinwoo Kim^2,*^, Hye-Jin Yoon^1^, Kyeong Sik Jin^3^, Sangryeol Ryu^2,4,†^ & Hyung Ho Lee^a,†^

^1^Department of Chemistry, College of Natural Sciences, Seoul National University, Seoul 08826, Korea

^2^Department of Food and Animal Biotechnology, Department of Agricultural Biotechnology, and Research Institute for Agriculture and Life Sciences, Seoul National University, Seoul 08826, Korea

^3^Pohang Accelerator Laboratory, Pohang University of Science and Technology, 80 Jigokro-127-beongil, Nam-Gu, Pohang, Kyungbuk 37673, Korea

^4^Center for Food and Bioconvergence, Seoul National University, Seoul 08826, Korea

^*^These authors contributed equally to this work.

^†^Corresponding author:

Professor Sangryeol Ryu, Department of Food and Animal Biotechnology, Department of Agricultural Biotechnology, and Research Institute for Agriculture and Life Sciences, Seoul National University, Seoul 08826, Korea

^†^Corresponding author:

Professor Hyung Ho Lee, Department of Chemistry, College of Natural Sciences, Seoul National University, Seoul 08826, Korea;

**Supplementary figures**

**
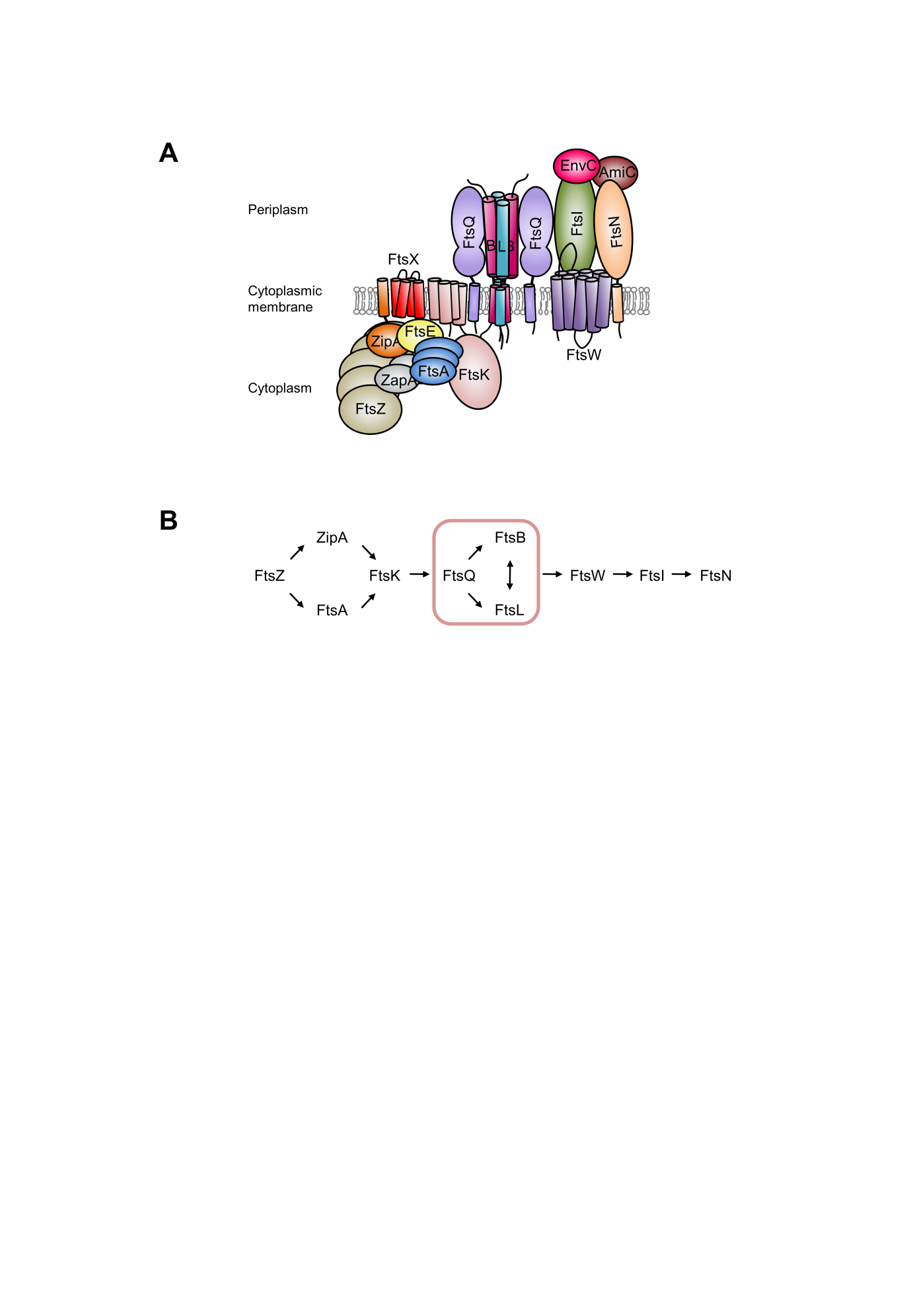
**

**Figure S1. Schematic overview of *E. coli* divisome complex.** (**A**) The key proteins (FtsE/X, FtsZ, FtsA, ZipA, ZapA, FtsK, FtsQ/B/L, FtsI/W, FtsN, AmiC, and EnvC) that form a structure known as the divisome are shown. (**B**) Schematic flow of divisome proteins recruited from FtsZ ring in *E. coli*.

**
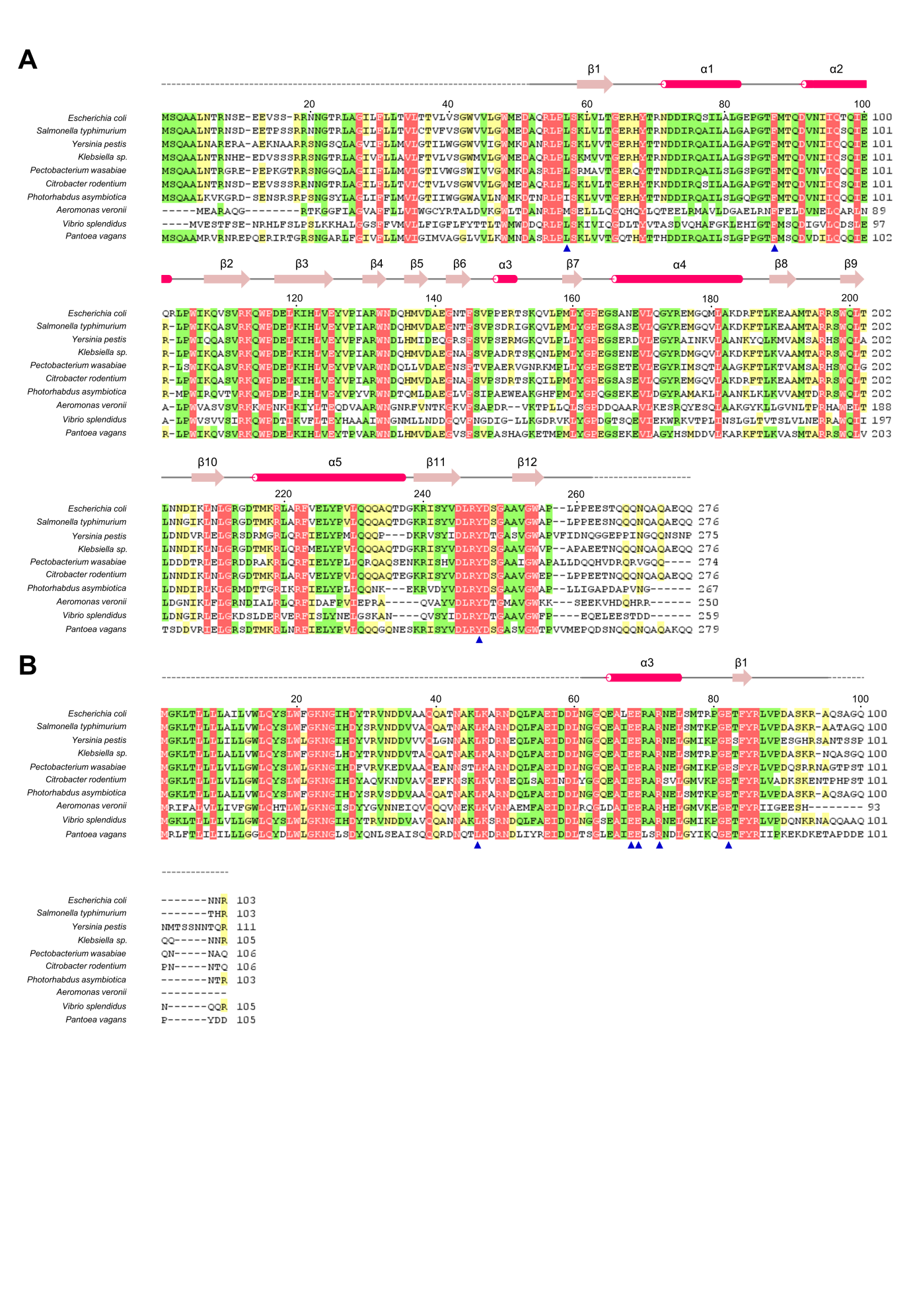
**

**Figure S2.** **Sequence alignments of FtsQ and FtsB.** (**A**) Multi-alignment of *E. coli* FtsQ (UniProt # P06136) against FtsQ from *Salmonella typhimurium* str. LT2 (UniProt # Q7CR81), *Yersinia pestis* (UniProt # Q7CGB1), *Klebsiella quasipneumoniae subsp. similipneumoniae* (UniProt # W8XR87), *Pectobacterium wasabiae* CFBP 3304 (UniProt # A0A1D7YY59), *Citrobacter rodentium* str. ICC168 (UniProt # D2TGN0), *Photorhabdus asymbiotica subsp. asymbiotica* str. ATCC 43949 (UniProt # C7BQ29), *Aeromonas veronii* AMC35 (UniProt # K1J1M1), *Vibrio splendidus* ATCC 33789 (UniProt # F9SHH9), and *Pantoea vagans* str. C9-1 (UniProt # E1SD94). (**B**) Multi-alignment of *E. coli* FtsB (UniProt # T1SA45) against FtsB from *S. typhimurium* str. LT2 (UniProt # Q7CR81), *Y. pestis* (UniProt # Q7CGB1), *K. quasipneumoniae subsp. similipneumoniae* (UniProt # W8XR87), *P. wasabiae* CFBP 3304 (UniProt #A0A1D7YY59), *C. rodentium* str. ICC168 (UniProt # D2TGN0), *P. asymbiotica subsp. asymbiotica* str. ATCC 43949 (UniProt # C7BQ29), *A. veronii* AMC35 (UniProt # K1J1M1), *V. splendidus* ATCC 33789 (UniProt # F9SHH9), and *P. vagans* str. C9-1 (UniProt # E1SD94). Secondary structure elements were assigned by PyMOL (The PyMOL Molecular Graphics System, http://www.pymol.org), and every twentieth residue is marked by a black dot. Strictly (100%) and semi-conserved residues (80% and 60%) are highlighted in red, green, and yellow, respectively. Cylinders and arrows above the sequences denote α-helices and β-strands, respectively. Blue triangles below the sequences indicate the mutation sites for FtsQ-FtsB binding study.

**
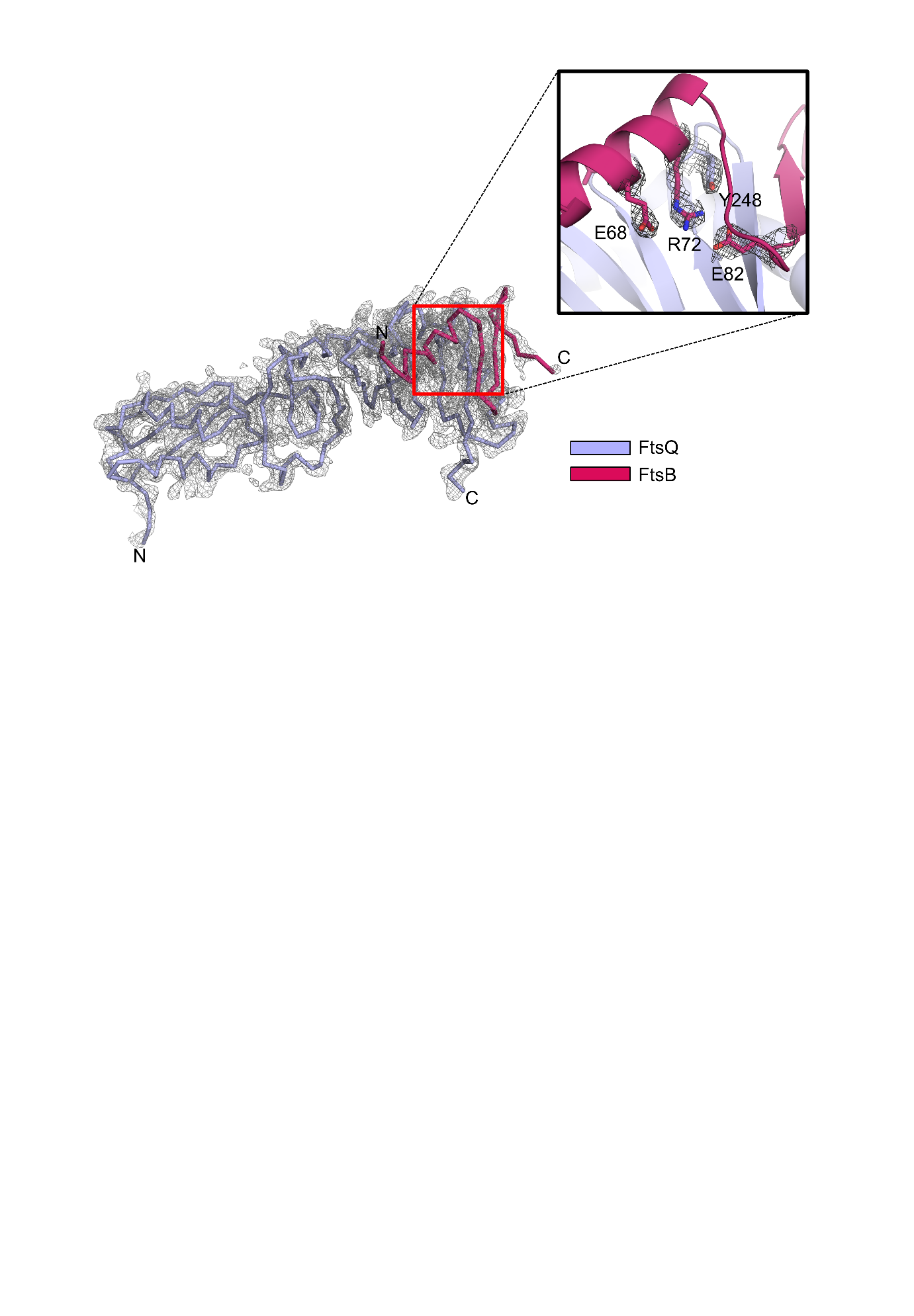
**

**Figure S3. Refined electron density (2Fo-Fc, 1.0 σ) of FtsQ_50-276_/FtsB_25-103_.** Each chain is shown in a different color, and magnified view of representative interaction region between FtsQ and FtsB is shown.

**
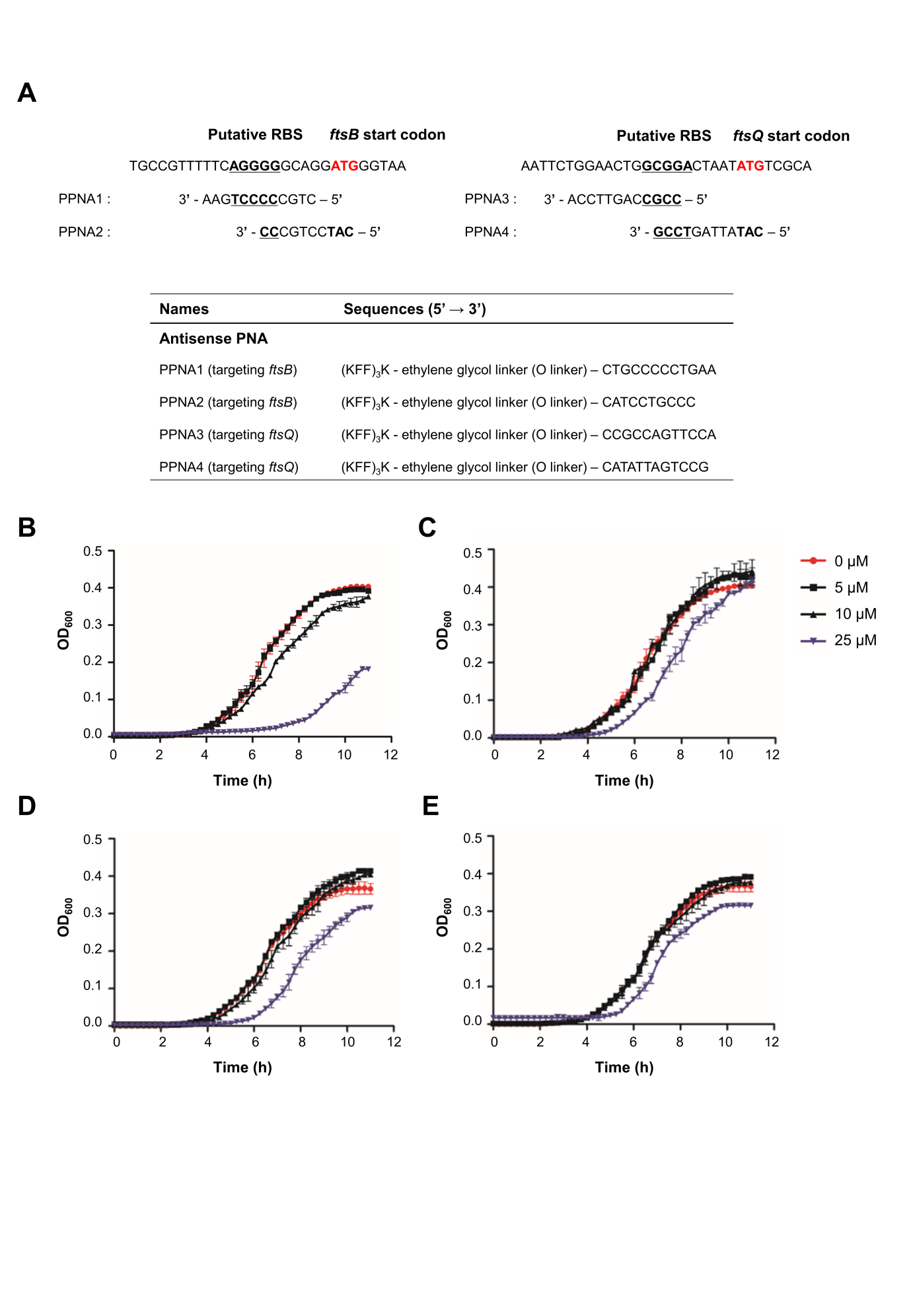
**

**Figure S4. Design and optimization of PPNA targeted to *ftsB* or *ftsQ* gene.** (**A**) Target sequence of (KFF)_3_K linked PNAs against *ftsB* or *ftsQ*. PPNAs were designed to be complementarily combined with start codon and ribosome binding site of *ftsB* or *ftsQ* gene. Growth curve of MG1655 treated with PPNA. Cells were prepared as described in *Materials and Methods*, and incubated with PPNA1 (**B**) PPNA2 (**C**) PPNA3 (**D**), and PPNA4 (**E**). Cells were monitored spectrophotometrically with a SpectraMax i3 Plus Microplate Spectrophotometer (Molecular Devices, Sunnyvale, CA, USA) by measuring absorbance at 600 nm (OD_600_). Data are presented as the mean ± SD of two independent experiments.


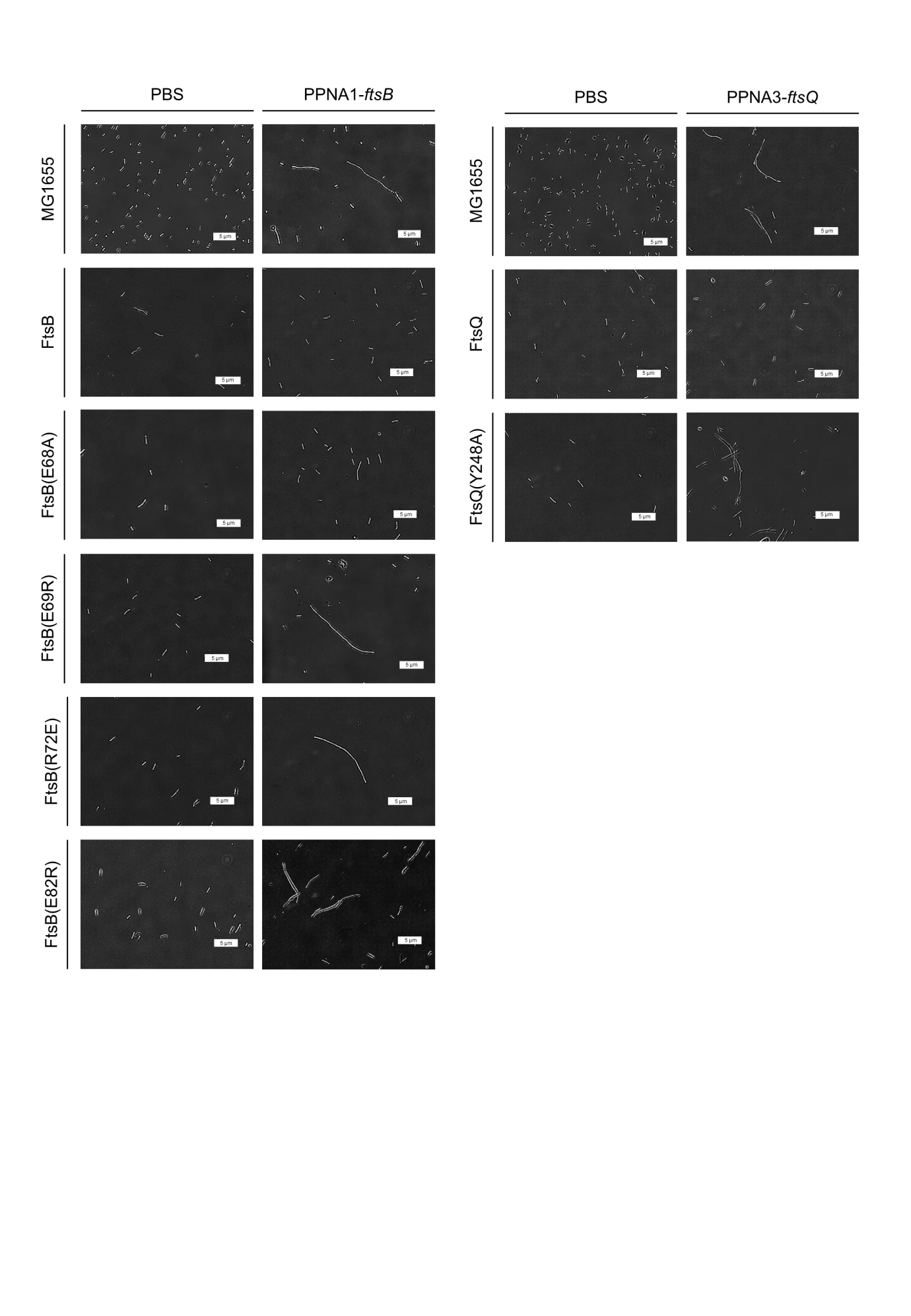


**Figure S5. Characterization of cell length when treated with PPNA.** The PPNAs lead to filamentation in cells expressing a mutated protein which has the inability to form the FtsQB complex. *E. coli* MG1655 strains harboring pftsB, pftsB (E68A), pftsB (E69R), pftsB (R72E), pftsB (E82R), pftsQ, and pftsQ (Y248A) were grown in LB medium containing 0.1 mM IPTG at 37 °C for 3 h. The cultures were diluted to 5 × 10^4^ CFU/mL and incubated with 12.5 μM anti-ftsB PPNA1 or anti-FtsQ PPNA3 at 37 °C for 9 h. Subsequently, samples were observed by epifluorescence microscopy (DE/Axio Imager A1 microscope, Carl Zeiss, Oberkochen, Germany).


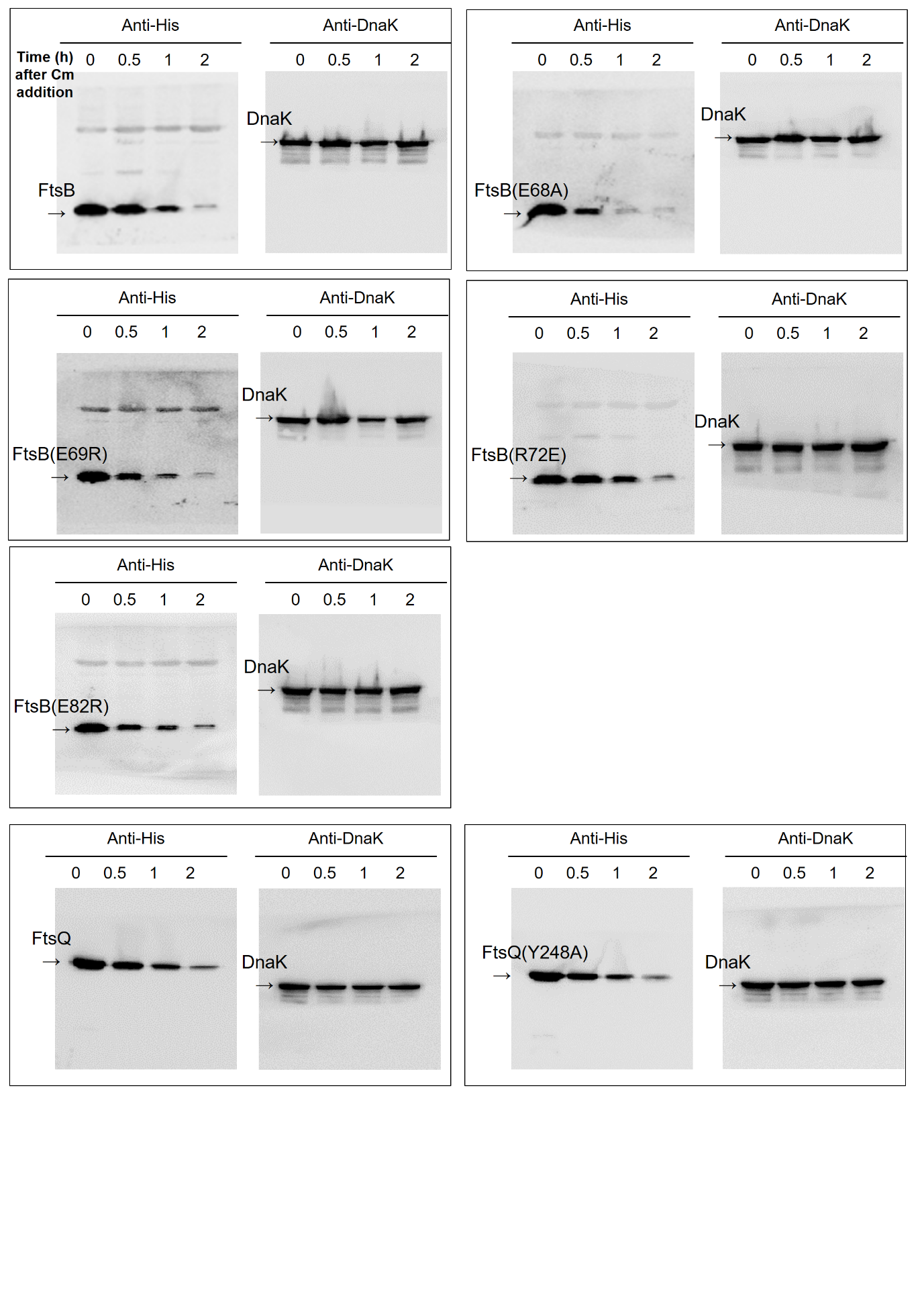


**Figure S6. Full-length western blot of Fig. 4B.** Comparison of the stability between WT and mutated proteins. *E. coli* MG1655 harboring plasmid pUHE21-2-*lacI^q^* encoding C-terminal His-tagged WT or mutated protein was sampled at the indicated time points after adding chloramphenicol and subjected to the Western blotting. DnaK was used as a loading control. The blots of target proteins were indicated with an arrow.

**
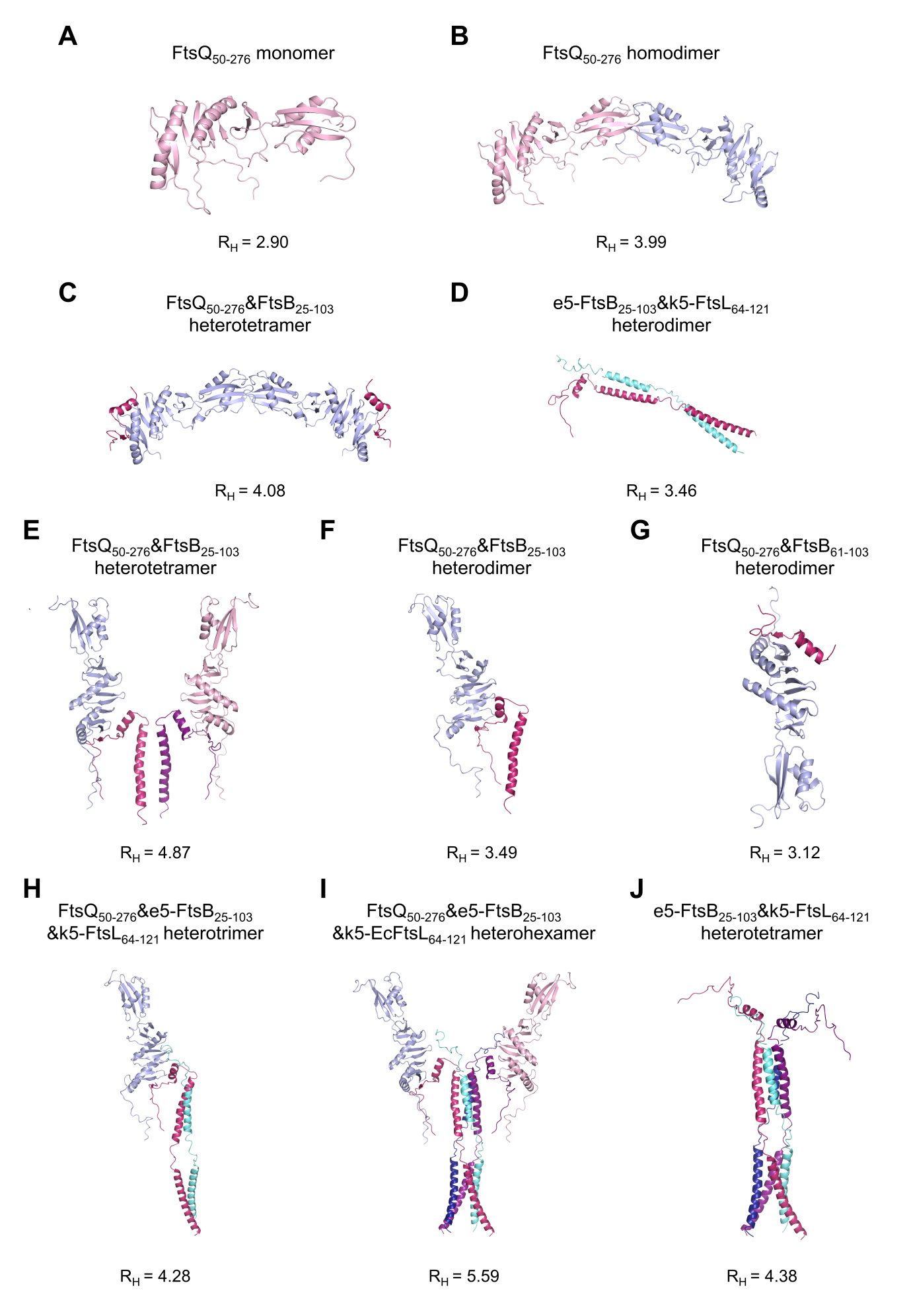
**

**Figure S7. Structure models and theoretical R_H_ values.** (**A-J**) Structure models for R_H_ calculation were generated based on the crystal structures of the FtsQ_50-276_-FtsB_25-103_ complex and SAXS solution structures of the FtsQBL complex, and were used for comparison with those of analytical gel filtration (Fig. 5B).

**
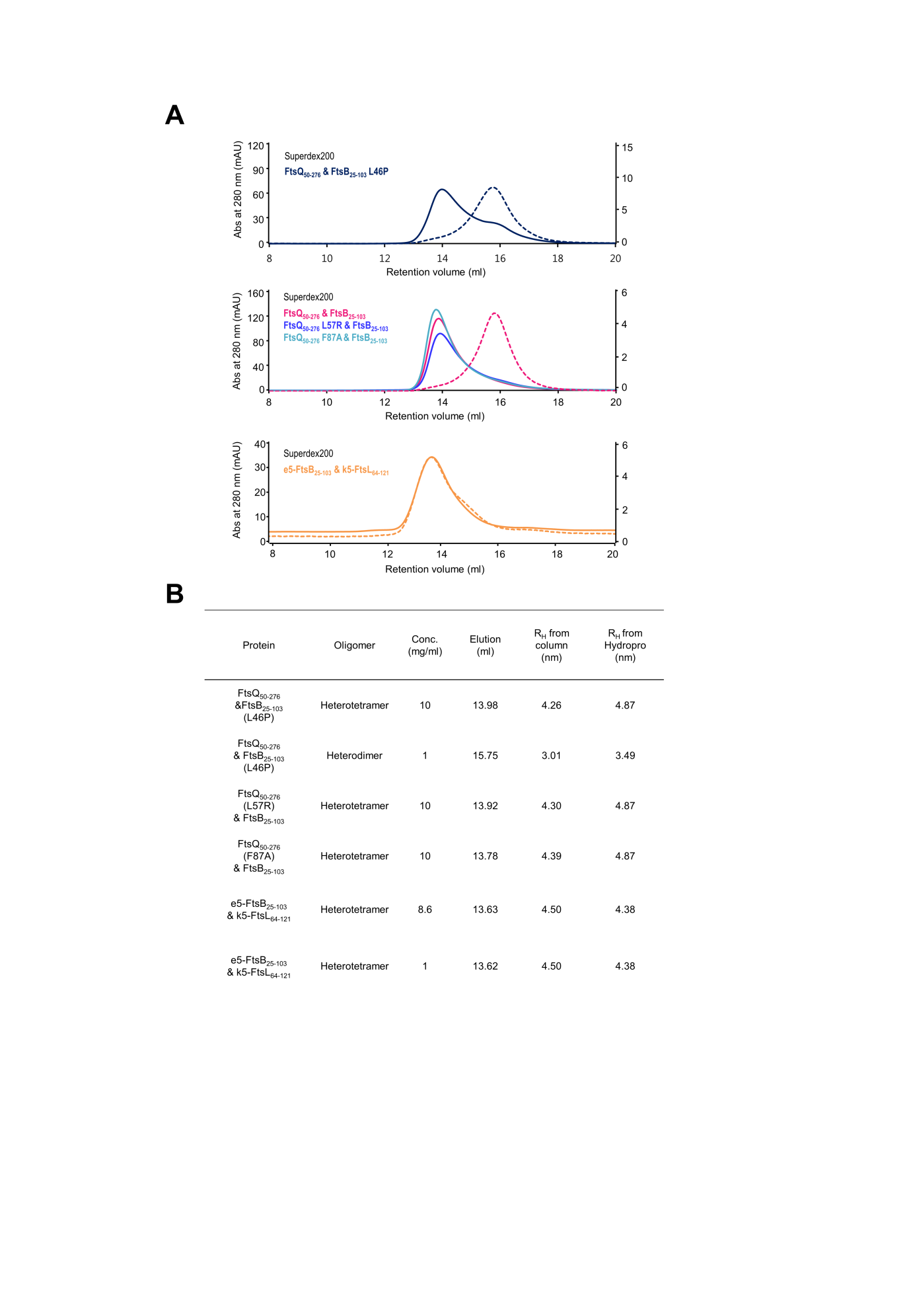
**

**Figure S8. Concentration-dependent oligomerization of FtsQ_50-276_/FtsB_25-103_ mutants (FtsB L46P, FtsQ L57R, and FtsQ F87A, respectively) and e5-FtsB_25-103_/k5-FtsL_64-121_ complex.** (**A**) Analytical gel filtration profiles of FtsQ_50-276_/FtsB_25-103_ L46P complex (navy), FtsQ_50-276_/FtsB_25-103_ complex (pink), FtsQ_50-276_ L57R/FtsB_25-103_ complex (blue), FtsQ_50-276_ F87A/FtsB_25-103_ complex (aquamarine), and e5-FtsB_25-103_/k5-FtsL_64-121_ complex (orange) at high (~10 mg/mL, solid lines and left y-axis) and low (1 mg/mL, dotted lines and right y-axis) concentrations. (**B**) Summary of the hydrodynamic analysis in Supplementary Fig. S8A. R_H_ values from column were compared to those calculated from structure models (Supplementary Fig. S7) using HYDROPRO program.


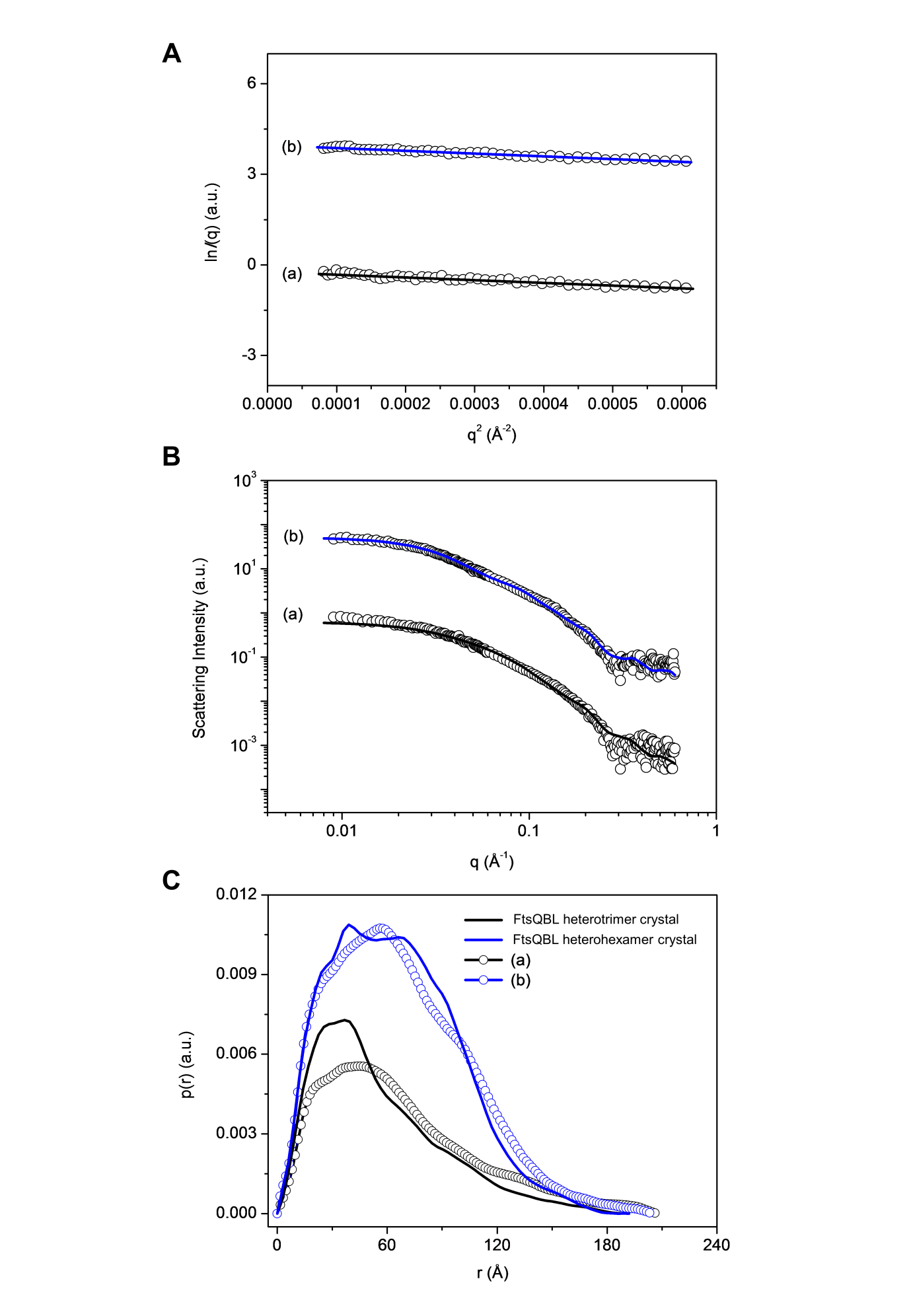


**Figure S9. Guinier plots, X-ray scattering profiles, and pair distance distribution *p*(*r*) functions of FtsQBL proteins.** (**A**) Guinier plots of the X-ray scattering profiles of FtsQBL proteins in aqueous solution. (a) FtsQBL heterotrimer, (b) FtsQBL heterohexamer. The straight lines were obtained from the linear regression of the scattering data in the *q*^2^ region. For clarity, each plot is shifted along the ln *I*(*q*) axis. (**B**) X-ray scattering profiles of FtsQBL proteins in solution, measured at 4 °C; (a) FtsQBL heterotrimer, (b) FtsQBL heterohexamer. The open symbols are the experimental data and solid lines are theoretical SAXS curve calculated from the predicted crystal structures of FtsQBL heterotrimer (χ^2^ = 0.449) and heterohexamer (χ^2^ = 0.112) proteins using the program CRYSOL. For clarity, each plot is shifted along the log *I(q)* axis. (**C**) The pair distance distribution *p*(*r*) functions for FtsQBL proteins in aqueous solution, based on an analysis of the experimental SAXS data using the program GNOM; (a) FtsQBL heterotrimer, (b) FtsQBL heterohexamer. The areas under the curves were normalized to the molecular weight.

**Supplementary tables**

**Table S1. Bacteria and plasmids used in this study.**

| **Strains** | | | **Characteristics** | | | **References** |
| --- | --- | --- | --- | --- | --- | --- |
| **Bacteria** | | | |  | |  |
| MG1655 | | | | | K-12 wild-type strain | Laboratory collection |
| BTH101 | | | | F^-^, *cyaA*-99, *ara*D139, *gal*E15, *gal*K16, *rps*L1, *hsd*R^2^, *mcr*A1, *mcr*B1 | 1 |  |
| BL21 (DE3) | | | | F^-^ *ompT* *hsdS_B_* (*r_B_^-^ m_B_^-^*) *gal dcm* (DE3) | Laboratory collection |  |
| **Plasmids** | | | | |  |  |
| pET21a | | | | Expression vector, rep_pMB1_ *lacI* Ap^R^, T7 promoter | Novagen |  |
| pRSF-duet | | | | Expression vector, rep_RSF1030_ *lacI* Km^R^, T7 promoter | Novagen |  |
| pGST2 | | | | Expression vector, rep_pMB1_ *lacI^q^* Ap^R^, tac promoter | 2 |  |
| pET21a-ftsQ | | | pET21a- *ftsQ*(50-276)-*his_x6_* (C-terminal His tag) | This study |  |  |
| pET21a-ftsQ(L57R) | | | pET21a- *ftsQ*(50-276) L57R-*his_x6_* | This study |  |  |
| pET21a-ftsQ(F87A) | | | pET21a- *ftsQ*(50-276) F87A-*his_x6_* | This study |  |  |
| pET21a-ftsQ(Y248A) | | | pET21a- *ftsQ*(50-276) Y248A-*his_x6_* | This study |  |  |
| pGST2-ftsB | | | pGST2-*GST*-*ftsB*(25-103) (N-terminal GST tag) | This study |  |  |
| pGST2-ftsB(L46P) | | | pGST2-*GST-ftsB*(25-103) L46P | This study |  |  |
| pGST2-ftsB(25-60) | | | pGST2-*GST-ftsB* (25-60) | This study |  |  |
| pGST2-ftsB(61-103) | | | pGST2-*GST-ftsB* (61-103) | This study |  |  |
| pGST2-e5-ftsB | | | pGST2-*GST-e5-FtsB*(25-103) | 3 |  |  |
| pGST2-e5-ftsB(E68A) | | | pGST2- *GST-e5-FtsB* (25-103) E68A | 3 |  |  |
| pGST2-e5-ftsB(E69R) | | | pGST2- *GST-e5-FtsB* (25-103) E69R | 3 |  |  |
| pGST2-e5-ftsB(R72E) | | | pGST2- *GST-e5-FtsB* (25-103) R72E | 3 |  |  |
| pGST2-e5-ftsB(E82R) | | | pGST2- *GST-e5-FtsB* (25-103) E82R | 3 |  |  |
| pRSF-k5-ftsL | | | pRSF-*his_x6_*-*k5-FtsL*(64-121) (N-terminal His tag) | 3 |  |  |

| pUHE21-2-*lacI^q^* | | rep_pMB1_ *lacI^q^* Ap^R^, P*_lac_* promoter | 4 |
| --- | --- | --- | --- |
| pftsB | pUHE21-2-*lacI^q –^ftsB* | This study |  |
| pftsB(E68A) | pUHE21-2-*lacI^q^-ftsB*(E68A) | This study |  |
| pftsB(E69R) | pUHE21-2-*lacI^q^-ftsB*(E69R) | This study |  |
| pftsB(R72E) | pUHE21-2-*lacI^q^-ftsB*(R72E) | This study |  |
| pftsB(E82R) | pUHE21-2-*lacI^q^-ftsB*(E82R) | This study |  |
| pftsQ | pUHE21-2-*lacI^q^-ftsQ* | This study |  |
| pftsQ(Y248A) | pUHE21-2-*lacI^q^-ftsQ*(Y248A) | This study |  |
| pftsB-*his_x6_* | pUHE21-2-*lacI^q^*-*ftsB*-*his_x6_* | This study |  |
| pftsB(E68A)-*his_x6_* | pUHE21-2-*lacI^q^*-*ftsB*(E68A)-*his_x6_* | This study |  |
| pftsB(E69R)-*his_x6_* | pUHE21-2-*lacI^q^*-*ftsB*(E69R)-*his_x6_* | This study |  |
| pftsB(R72E)-*his_x6_* | pUHE21-2-*lacI^q^*-*ftsB*(R72E)-*his_x6_* | This study |  |
| pftsB(E82R)-*his_x6_* | pUHE21-2*-lacI^q^*-*ftsB*(E82R)-*his_x6_* | This study |  |
| pftsQ-*his_x6_* | pUHE21-2-lacIq-*ftsQ*-*his_x6_* (C-terminal His tag) | This study |  |
| pftsQ(Y248A)-*his_x6_* | pUHE21-2*-lacI^q^*-*ftsQ*(Y248A)-*his_x6_* | This study |  |
| pKT25 | | p15A *ori*, *Plac::cyaA* 1-224, Km^R^ | 1 |
| pKT25-zip | | pKT25-zip (T25-Zip, T25 domain of Cya fused with leucine zipper of GCN4, C-terminal) | 1 |
| pKT25-ftsQ | | pKT25-*ftsQ* (T25-FtsQ) | This study |
| pKT25-ftsQ(Y248A) | | pKT25-*ftsQ*(Y248A) (T25-FtsQ(Y248A)) | This study |
| pUT18C | | ColEI *ori*, *Plac::cyaA* 225–399, Ap^R^ | 1 |
| pUT18C-zip | pUT18C-zip (T18-Zip, T18 domain of Cya fused with leucine zipper of GCN4, C-terminal) | 1 |  |
| pUT18C-ftsB | pUT18C-*ftsB* (T18-FtsB) | This study |  |
| pUT18C-ftsB(E68A) | pUT18C-*ftsB*(E68A) (T18-FtsB(E68A)) | This study |  |
| pUT18C-ftsB(E69R) | pUT18C-*ftsB*(E69R) (T18-FtsB(E69R)) | This study |  |
| pUT18C-ftsB(R72E) | pUT18C-*ftsB*(R72E) (T18-FtsB(R72E)) | This study |  |
| pUT18C-ftsB(E82R) | pUT18C-*ftsB*(E82R) (T18-FtsB(E82R)) | This study |  |

| Ap^R^, ampicillin resistant; Km^R^, kanamycin resistant; |
| --- |

**Table S2. Primers used for the construction of bacterial strains and plasmids.**

| **Primers** | **Sequences (5’ to 3’)** |
| --- | --- |
| FtsQ(50-276)-F | GCT ATA TGC TAG CGA AGA TGC GCA ACG CCT GCC |
| FtsQ(50-276)-R | GCT AAT TCT CGA GTT GTT GTT CTG CCT GTG CCT GAT |
| FtsQ(50-276)-Y248A-F | TTA GCT ACG TTG ATT TCG GTG CAG ACT CTG GAG CGG CAG TAG G |
| FtsQ(50-276)-Y248A-R | CCT ACT GCC GCT CCA GAG TCT GCA CGC AAA TCA ACG TAG CTA A |
| FtsQ(50-276)-L57R-F | AAG ATG CGC AAC GCC TGA CGC GTT CAA AGC TGG TGT TGA CCG G |
| FtsQ(50-276)-L57R-R | CCG GTC AAC ACC AGC TTT GAA CGC GGC AGG CGT TGC GCA TCT T |
| FtsQ(50-276)-F87A-F | CAT TGG GTG AGC CGG GTA CCG CGA TGA CCC AGG ATG TCA ACA T |
| FtsQ(50-276)-F87A-R | ATG TTG ACA TCC TGG GTC ATC GAG GTA CCC GGC TCA CCC AAT G |
| FtsB(25-60)-F | GCT ATA TCC ATG GGA GGT ATA CAT GAC TAT ACC CGC GT |
| FtsB(25-60)-R | GCT AAT TCT CGA GTC AGA GAT CGT CAA TTT CGG CAA AAA G |
| FtsB(61-103)-F | GCT ATA TCC ATG GGA AAT GGC GGC CAG GAG GCG CT |
| FtsB(61-103)-R | GCT AAT TCT CGA GTT ATC GAT TGT TTT GCC CCG CAG A |
| e5-FtsB(25-103)-F | GCT ATA TCC ATG GGA GCT AGC GAG GTA AGC GCC CT |
| e5-FtsB(25-103)-R | GCT AAT TCT CGA GTT AGC GAT TGT TTT GGC CCG CAG A |
| FtsB(25-103)-L46P-F | CAA TGA TGA TGT GGC GGC ACA GCA AGC TAC AAA CGC GAA ACC GAA AGC GCG AAA CGA TCA ACT TTT TGC CGA AAT TGA CGA TC |
| FtsB(25-103)-L46P-R | GAT CGT CAA TTT CGG CAA AAA GTT GAT CGT TTC GAG CTT TCG GTT TCG CGT TTG TAG CTT GCT GTG CCG CCA CAT CAT CAT TG |
| e5-FtsB(25-103)-F | GCT ATA TCC ATG GGA GCT AGC GAG GTA AGC GCC CT |
| e5-FtsB(25-103)-R | GCT AAT TCT CGA GTT AGC GAT TGT TTT GGC CCG CAG A |
| e5-FtsB(25-103)-E68A-F | ATG GCG GCC AGG AGG CGC TCG CAG AGC GTG CGC GTA ATG AAC T |
| e5-FtsB(25-103)-E68A-R | AGT TCA TTA CGC GCA CGC TCT GCG AGC GCC TCC TGG CCG CCA T |
| e5-FtsB(25-103)-E69R-F | GCG GCC AGG AGG CGC TCG AAA GAC GTG CGC GTA ATG AAC TCA G |
| e5-FtsB(25-103)-E69R-R | CTG AGT TCA TTA CGC GCA CGT CTT TCG AGC GCC TCC TGG CCG C |
| e5-FtsB(25-103)-R72E-F | AGG CGC TCG AAG AGC GTG CGG AAA ATG AAC TCA GCA TGA CCA G |
| e5-FtsB(25-103)-R72E-R | CTG GTC ATG CTG AGT TCA TTT TCC GCA CGC TCT TCG AGC GCC T |
| e5-FtsB(25-103)-E82R-F | TCA GCA TGA CCA GGC CGG GCA GAA CTT TTT ATC GTC TGG TGC C |
| e5-FtsB(25-103)-E82R-R | GGC ACC AGA CGA TAA AAA GTT CTG CCC GGC CTG GTC ATG CTG A |
| k5-FtsL(64-121)-F | GCT ATA TGG ATC CGG AAA ACC TGT ATT TTC AGG GCT CGC GTA AGG TAT CTG CTT TAA AG |
| k5-FtsL(64-121)-R | GCT AAT TGT CGA CTT ATT TTT GCA CTA CGA TAT TTT CTT GTG |

**Table S3. Structural parameters obtained from the SAXS data of FtsQBL heterotrimer and heterohexamer in solution.**

| Sample | *R*_g,G_^a^ (Å) | *R*_g,p(r)_^b^ (Å) | *D*_max_^c^ (Å) | *MM*_calculated_^d^ (kDa) | *MM*_SAXS_^e^ (kDa) |
| --- | --- | --- | --- | --- | --- |
| Trimeric model | 44.0 ± 0.2 | 46.1 ± 2.4 | 192.0 | 49.7 | - |
| Hexameric model | 50.1 ± 0.1 | 51.1 ± 1.3 | 192.0 | 99.4 | - |
| (a) FtsQBL heterotrimer | 51.2 ± 4.0 | 54.3 ± 2.8 | 207 | 49.7 | 62.5 |
| (b) FtsQBL  heterohexamer | 51.3 ± 1.9 | 54.1 ± 2.3 | 203 | 99.4 | 97.9 |

^a^*R*_g,G_ (radius of gyration) was obtained from the scattering data by the Guinier analysis.

^b^*R*_g,p(r)_ (radius of gyration) was obtained from the *p*(*r*) function by the program GNOM.

^c^*D*_max_ (maximum dimension) was obtained from the *p*(*r*) function by the program GNOM.

^d^*MM*_calculated_ (molecular mass) was obtained from the amino acid sequence of protein.

^e^*MM*_SAXS_ (molecular mass) was estimated from a BSA standard protein.

References

1. Karimova, G., Pidoux, J., Ullmann, A. & Ladant, D. A bacterial two-hybrid system based on a reconstituted signal transduction pathway. *Proc. Natl. Acad. Sci.* USA **95**, 5752-5756 (1998).

2. Sheffield, P., Garrard, S. & Derewenda, Z. Overcoming expression and purification problems of RhoGDI using a family of "parallel" expression vectors. *Protein Expr. Purif.* **15**, 34-39 (1999).

3. Glas, M. *et al.* The soluble periplasmic domains of *Escherichia coli* cell division proteins FtsQ/FtsB/FtsL form a trimeric complex with submicromolar affinity. *J. Biol. Chem.* **290**, 21498-21509 (2015).

4. Soncini, F. C., Vescovi, E. G. & Groisman, E. A. Transcriptional autoregulation of the *Salmonella* *Typhimurium* PhoPQ operon. *J. Bacteriol.* **177**, 4364-4371 (1995).
